# Regulating periodontal disease with smart stimuli-responsive systems: Antimicrobial activity, immunomodulation, periodontium regeneration

**DOI:** 10.1016/j.mtbio.2025.101863

**Published:** 2025-05-15

**Authors:** Guang-Liang Su, Yu-Jun Peng, Hong-Ze Ruan, Juan Cheng, Tian Deng, Yu-Feng Zhang

**Affiliations:** a State Key Laboratory of Oral & Maxillofacial Reconstruction and Regeneration, Key Laboratory of Oral Biomedicine Ministry of Education, Hubei Key Laboratory of Stomatology, School & Hospital of Stomatology, Taikang Center for Life and Medical Sciences, Wuhan University, Wuhan, 430079, PR China; b Medical Research Institute, School of Medicine, Wuhan University, Wuhan, 430071, PR China; c Department of Stomatology, Zhongnan Hospital of Wuhan University, PR China

**Keywords:** Periodontal disease, Stimuli-responsive, Antimicrobial activity, Immunomodulation, Periodontium regeneration

## Abstract

Periodontal disease is a worldwide inflammatory condition that seriously affects both oral and systemic health. The presence of microbial biofilms and the dysregulation of the host immune response are considered crucial factors in the initiation and progression of periodontal disease. Mechanical debridement combined with antibiotic therapy is the standard non-surgical treatment for periodontal disease; however, this approach faces limitations in deep bacterial clearance and resistance to antibiotics. Although some new drugs and accessible nanodelivery systems have been developed, their targeting accuracy and drug utilization still require improvement in the complex oral environment. In recent years, intelligent biomaterials with stimuli-responsive characteristics have garnered more attention due to their unique advantages. These materials can respond to specific physiological signals or external stimuli, enabling precise release of functional agents. However, existing studies focus on the optimization of the single material system, lacking the horizontal comparisons and clinical evaluations of different stimulus-responsive materials. This review aims to address this gap by systematically examining the roles of endogenous and exogenous stimuli in regulating the periodontal disease progression and activating responsive substances. While various stimulus-regulated systems have their respective advantages, the complex oral environment necessitates synergistic action among multiple signals. The review further explores the applications of smart responsive materials in eradicating periodontal pathogens, regulating the inflammatory microenvironment, and promoting periodontium regeneration. Coordinated integration of functional mechanisms is crucial to achieving periodontal disease recovery. Moreover, the challenges faced by intelligent responsive materials in periodontal disease treatment are examined, along with outlining potential directions for future research. It outlines potential research directions to prioritize personalized material design, safety evaluations, and production quality control to advance clinical application.

## Graphical abstract

**Figure:**
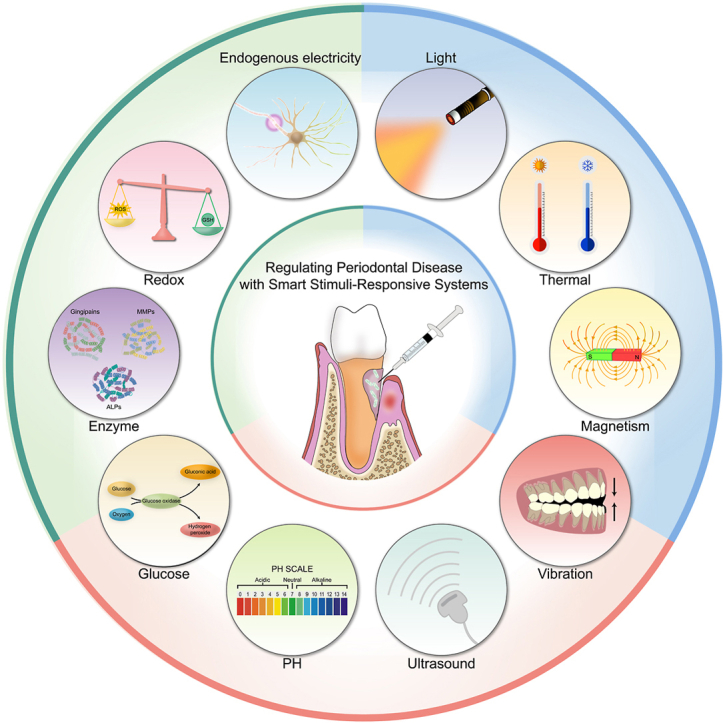

## 1 Introduction

Periodontal disease is recognized by the World Health Organization (WHO) as a significant oral health issue and a public health concern affecting over 19 % of the global adult population. [1] Periodontal diseases, including periodontitis, gingivitis, and peri-implantitis, are characterized by chronic inflammation of periodontium, which can cause damage to these tissues and ultimately result in tooth loss. [2] Furthermore, a bibliometric analysis of 1,519 publications from 1989 to 2024 reveals that periodontitis exhibits robust systemic associations, including metabolic and nutritional disorders, cardiovascular diseases, female reproductive and pregnancy-related pathologies, as well as musculoskeletal diseases. [3] The etiology of periodontal disease is complex, with the presence of microbial biofilms and dysregulation of the host immune response considered key factors in the initiation and progression of the disease. [4] Non-surgical therapies, including mechanical debridement, such as scaling and root planning, and adjunctive antibiotic treatment, aim to eliminate plaque biofilm to control periodontal disease. Studies indicate that periodontal therapies also improve systemic outcomes in diabetes, cardiovascular diseases, and adverse pregnancy outcomes. [[5], [6], [7], [8]] However, conventional periodontal therapies still face significant limitations. Mechanical debridement exhibits restricted efficacy in eliminating bacteria within deep periodontal pockets and is highly operator-dependent. [9] Moreover, the global overuse of antibiotics has exacerbated antimicrobial resistance. A retrospective study of 7,804 periodontitis patients revealed that 63.5 % of cases developed resistance to at least one antibiotic. Notably, antibiotic resistance rates surged from 37 % in 2008 to 70 % in 2015. [10] On the other hand, the global economic burden of periodontal diseases cannot be overlooked. According to the World Health Organization (WHO), direct expenditures for global oral diseases reached 387 billion dollars in 2019. [11] The S3-level clinical guidelines for periodontitis treatment from the European Federation of Periodontology further indicate that periodontitis alone is estimated to cost 54 billion dollars in direct treatment costs and 25 billion dollars in indirect costs. [12] Given these challenges, developing advanced therapeutic approaches for periodontitis is necessary to enhance treatment efficacy and reduce public health expenditures.

Advances in materials science and biomedical engineering have led to the emergence of various drug delivery systems based on nanomaterials, including nanoparticles (NPs), hydrogels, films, and fibers. These systems offer new possibilities for the treatment of periodontal disease. [13] Nonetheless, the complexity of the oral environment poses challenges to therapy, and traditional nanomaterials still exhibit deficiencies in terms of targeting and drug utilization efficiency. In recent years, functional biomaterials with stimuli-responsive characteristics have demonstrated unique advantages in the treatment of periodontal disease. The core feature of these materials is their ability to respond to specific endogenous physiological signals, such as pH level, glucose concentration, enzyme activity, redox state, and intrinsic bioelectricity, as well as exogenous stimuli, including light, thermals, magnetic fields, vibrations, and ultrasound. This responsiveness enables the materials to release drugs or bioactive molecules at precise times and locations, facilitating targeted therapy for periodontal disease. As research continues to advance, significant progress has been made not only in the design and fabrication of these materials but also in showcasing their substantial potential in clinical applications. These intelligent biomaterials are progressively overcoming the limitations of existing technologies, paving new ways for the treatment of periodontal disease.

**Fig. 1:**
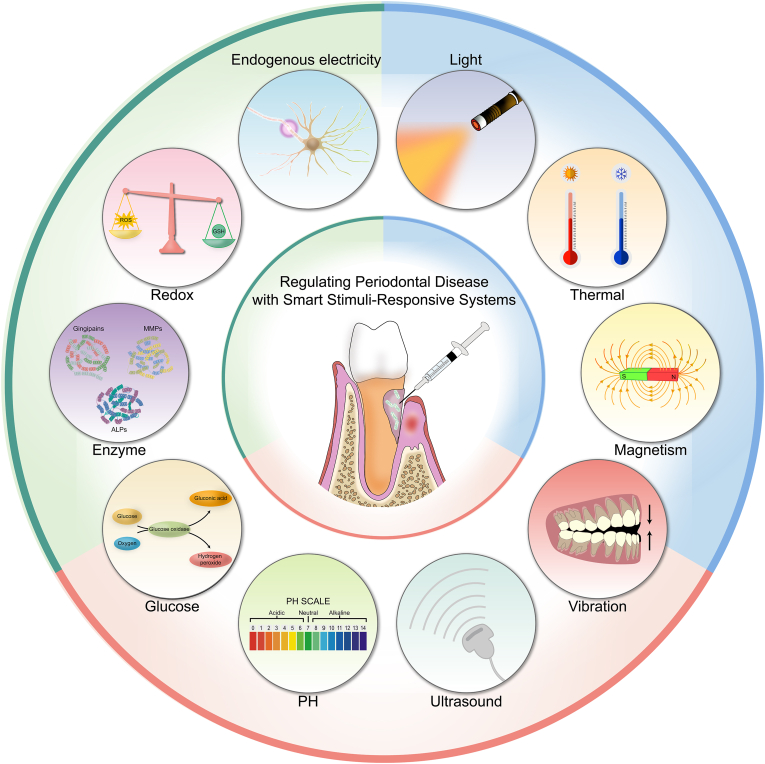
Regulating periodontal disease with smart stimuli-responsive systems. Smart responsive materials possess the capability to react precisely to specific endogenous or exogenous stimuli, enabling targeted delivery of active molecules. Endogenous stimuli include changes in pH, increased glucose concentration, upregulated enzyme expression, elevated redox potential, and bioelectric signals, whereas exogenous stimuli encompass light, thermal energy, magnetic fields, mechanical vibrations, and ultrasound.

However, current research predominantly focuses on optimizing the performance of single-stimulus-responsive systems, with limited exploration of comparative clinical evaluations across diverse stimuli-responsive mechanisms. To solve this problem and compensate for existing deficiencies, this review specifically investigates the application potential of smart responsive materials in periodontal therapy. This review conducted a comprehensive literature search across three databases (PubMed, Clarivate-Web of Science, and Embase) from their inception to December 31, 2024. The inclusion criteria encompassed original research articles, reviews, and preclinical/clinical trial reports, while excluding meeting abstracts and case reports. The study focus was centered on the application of "smart responsive materials" in "biomedical fields (particularly periodontal disease treatment)", including antimicrobial, anti-inflammatory, and tissue repair applications. Excluded were studies solely investigating conventional periodontal therapies (*e.g.* scaling, root planing, and surgical interventions) or non-responsive materials (*e.g.* conventional antibiotics, standard hydrogels). This review provides an analysis of the roles of endogenous and exogenous stimuli in modulating periodontal disease progression and activating responsive materials, emphasizing the necessity of multi-stimulus synergy (Fig. 1). Furthermore, this review systematically examines the application mechanisms of smart responsive materials in eradicating periodontal pathogens, regulating inflammatory microenvironments, and promoting periodontium regeneration. The recovery of periodontal disease relies on the multifunctional coordination of antibacterial, anti-inflammatory and tissue regeneration effects. This review also thoroughly examines the challenges associated with the application of smart responsive materials, including personalized adaptability in material design, long-term safety, and quality control in large-scale manufacturing. It emphasizes the need for future research to focus on these aspects to drive the clinical translation of this technology.

## 2 Physiological stimuli to mediate periodontal disease

The microenvironment of periodontal inflammation represents a critical target for therapeutic interventions, characterized by unique pathological traits such as acidic pH levels, accumulation of glucose, overexpression of enzymes, elevated redox potential, and endogenous bioelectric activity. [[14], [15], [16], [17], [18]] Intelligent responsive materials can react to the specific pathological microenvironment, targeting inflammatory sites to enhance local therapeutic efficacy while minimizing systemic side effects.

### 2.1 PH responsive materials

PH-responsive materials are intelligent systems that exhibit specific physicochemical changes (*e.g.* swelling, degradation, and charge reversal) in response to hydrogen ion (H^+^) concentration variations in their microenvironment. [19] The responsive mechanisms, which arise from protonation/deprotonation reactions of functional groups (*e.g.* amino, carboxyl groups, and pH-sensitive linkages) or cleavage of acid-labile bonds, enable targeted drug release through this process. [[20], [21], [22], [23]] The presence of pathogenic acidogenic bacteria can lower the local pH environment of the periodontal region to below 5.0, a significant deviation from the near-neutral pH levels observed under healthy conditions. [14] This pathological pH gradient difference enables pH-responsive materials to achieve precise controlled drug release in targeted therapy for periodontitis.

**Fig. 2:**
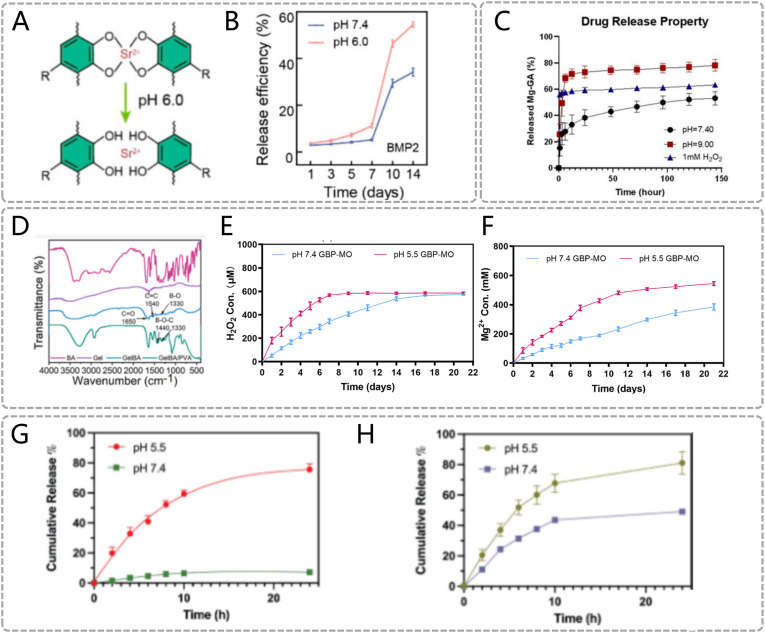
PH responsive materials. (A) pH-responsive releasing mechanism of Au@MPN@BMP2. (B) BMP2 release profiles of Au@MPN-BMP2 under different pH environments. (A, B) Reproduced with permission. [20] Copyright 2024, American Chemical Society. (C) The Mg-GA releasing profile of CSBDX@10MOF in different condition. Reproduced with permission. [21] Copyright 2024, The Author(s). (D) Fourier-transform infrared test of GelBA. (E) Release behavior of H_2_O_2_ and (F)Mg^2+^ at different pH values. (D, E, F) Reproduced with permission. [22] Copyright 2024, American Chemical Society. (G)In vitro release studies of TCS and (H)DFO under PH5.5 and PH7.4. (G, H) Reproduced with permission. [23] Copyright 2025, The Author(s).

The development of such biomaterials follows two primary strategies. The first involves employing polyelectrolytes that can undergo protonation or deprotonation in response to pH changes, such as materials with amine or carboxyl functional groups. These materials include lipids-derived products, chitosan (CS) nanostructures, and nanogels or microgels, which respond through conformational changes in acidic environments. For instance, a metal-phenolic nanocomposite Au@MPN-BMP2 promote effective periodontitis treatment through dual antimicrobial and osteogenic functions. [20] The phenolic hydroxyl groups of tannic acid (TA) coordinate with Sr^2+^ to form the metal-phenolic network (MPN) coating. Under low pH conditions, protonation of phenolic hydroxyl groups weakens their metal-coordination capacity, thereby loosening the network structure and triggering bone morphogenetic protein-2 (BMP2) release (Fig. 2A). Quantitative analysis demonstrates 54.5 ± 0.9 % cumulative BMP2 release at pH 6.0 versus 34.2 ± 1.4 % at pH 7.4 over 14 days (Fig. 2B). Released BMP2 activates bone marrow mesenchymal stem cells (BMSCs), enhancing osteogenic differentiation. [24] Additionally, TA in the material matrix interacts with bacterial membranes, disrupts membrane integrity, and induces bacterial death. Notably, periodontal pathogens (such as *Filifactoralocis, F.alocis*) decompose proteins to produce substances including amino acids and urea, which may increase the pH of periodontal pockets. [25] Accordingly, deprotonation-responsive strategies have been developed to control drug release in alkaline periodontal microenvironments. For example, a dynamic hydrogel-metal-organic framework system named CMCS/4-FPBA/DEX (CSBDX) promotes periodontal bone regeneration by controlled drug delivery. [21] Carboxymethyl chitosan (CMCS) and 4-formylphenylboronic acid (4-FPBA) form imine bonds through condensation reactions. Under alkaline conditions, deprotonation of imine bonds induces dynamic bond cleavage and releases Mg-GA Metal–Organic Framework (MOF). Drug release experiments show that the cumulative release of Mg-GA MOF under high pH conditions (74.43 %) is significantly higher than that in neutral environments (53.13 %) (Fig. 2C). The released Mg-GA MOF exerts multifunctional effects including antibacterial, antioxidant, and osteogenic activities, precisely regulating periodontal disease progression.

The second strategy entails integrating acid-labile bonds into the design of biomaterials. Such bonds, including ester, acetal, and ketone linkages, are unstable under acidic conditions, leading to material degradation. For example, a pH-responsive composite hydrogel GelBA/PVA/MgO_2_ is used to control peri-implant infection and repair infectious bone defects. [22] benzeneboronic acid (BA) forms phenylboronate ester bonds with the vicinal diol groups of polyvinyl alcohol (PVA) (Fig. 2D). Under acidic conditions, hydrolysis of ester bonds causes gel network dissociation and MgO_2_ release. MgO_2_ further decomposes into Mg^2+^ and H_2_O_2_ under acidic environments. Mg^2+^ and H_2_O_2_ synergistically inhibit bacterial growth and accelerate bone regeneration. In vitro experiments show that the total release amount and rate of GelBA/PVA/MgO_2_ under acidic conditions are significantly higher than those in neutral environments, enabling more effective functional execution (Fig. 2E) (Fig. 2F). Additionally, Ma et al. designed a pH-responsive nanogel NG-TCS-DFO for periodontitis treatment via antibacterial and pro-angiogenic effects. [23] The linear polyglycerol (IPG) backbone is linked to diethylamine (DEA) through acetal bonds. Triclosan (TCS) is conjugated with vinyl ether acrylate (VEA) via acetal bonds. The IPG-DEA backbone and TCS-VEA conjugates are covalently crosslinked to form a stable nanogel network (NG). Deferoxamine (DFO) is physically encapsulated within the hydrogel. TCS, a broad-spectrum antimicrobial agent, kills periodontal pathogens. DFO promotes angiogenesis and accelerates bone regeneration. Under acidic conditions, hydrolysis of acetal bonds triggers network disintegration and graded drug release. In vitro experiments demonstrate that the crosslinked structure is first disrupted under acidity, releasing 72 % of DFO within 24 h, followed by acetal bond cleavage releasing 78 % of TCS (Fig. 2G) (Fig. 2H).

As a fundamental endogenous stimulus, pH exhibits significant potential in regulating the response of materials used for periodontal disease treatment, yet it is also subject to inherent limitations. The pH levels in the periodontal environment can vary widely due to factors such as diet, salivary flow and microbial metabolism. [[25], [26], [27]] This variability in the pH environment can lead to unpredictable drug release patterns, thereby complicating the regulation and control of therapeutic outcomes.

### 2.2 Glucose responsive materials

Glucose-responsive materials are a class of polymer or nanomaterials that can sense glucose concentration changes and respond dynamically. [28] Their core mechanism relies on enzymatic catalysis or chemical structural responses. For example, glucose oxidase (GOx) catalyzes the conversion of glucose into gluconic acid and hydrogen peroxide (H_2_O_2_), thereby triggering material degradation or providing substrates for subsequent reactions. [29,30] Chemical response mechanisms utilize the reversible binding between glucose and specific groups, such as phenylboronic acid (PBA), to control drug release. [31,32] Diabetic periodontitis, is characterized by a unique microenvironment with accumulated hyperglycemia, elevated blood glucose levels, excessive accumulation of reactive oxygen species (ROS), and amplified inflammatory responses. [33] This pathological microenvironment activates the functional responses of glucose-responsive materials. The latter synchronously intervenes in ROS burst, chronic inflammation, and pathogenic biofilm formation by scavenging excess glucose, releasing antimicrobial agents, and modulating immune responses.

**Fig. 3:**
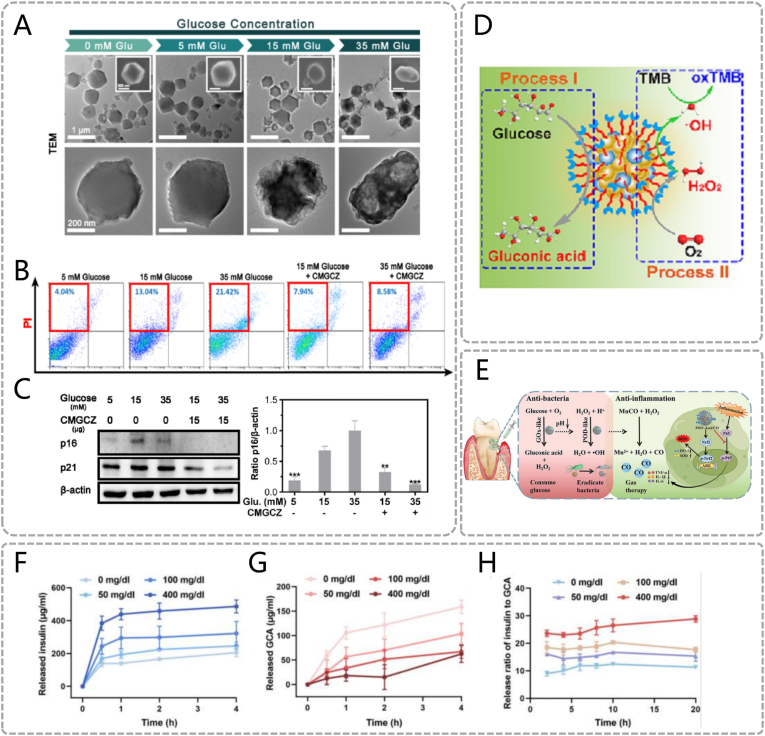
Glucose responsive materials. (A) Transmission electron microscope (TEM) images of CMGCZ under 0, 5, 15, and 35 mM glucose. (B) The pyroptosis of RAW264.7 cells under various conditions was detected via flow cytometric analysis. (C) Western Blotting assay revealed the expression levels of p16, p21 and β - actin in HGFs which had been cultured in the supernatant of RAW264.7's different medium for 24 h (A, B, C) Reproduced with permission. [29] Copyright 2023, The Author(s). (D) Diagrammatic illustration depicting the cascade reaction process of Au/Pt NCs@GOx. Reproduced with permission. [30] Copyright 2023, Elsevier. (E) Schematic representation of the cascade reaction mechanism for MSN-Au@CO. Reproduced with permission. [34] Copyright 2024, The Author(s). (F) The in - vitro cumulative insulin release from the GRI - MN patch at different glucose concentrations. (G) The in - vitro cumulative release of GCA from the GRG - MN patch at different glucose concentrations. (H) The release ratio of insulin to GCA from GRD-MN under different glucose concentrations. (F, G, H) Reproduced with permission. [32] Copyright 2022, The Author(s).

Currently, glucose-responsive materials for periodontal diseases are primarily driven by GOx catalytic mechanisms. These materials share structural similarities with pH-responsive systems, enabling drug-specific release in response to acidic products generated by GOx catalysis. For example, Tang et al. designed a glucose-driven switchable composite CaAlg@MINO/GOx/CAT/ZIF-8 (CMGCZ) for diabetic periodontitis treatment. [29] GOx catalyzes glucose oxidation to produce gluconic acid. The acid protonates Zn^2+^ coordination bonds in zeolitic imidazole framework-8 (ZIF-8), triggering structural collapse and minocycline (MINO) release (Fig. 3A). In vitro antibacterial assays show glucose concentration-dependent MINO release: CMGCZ releases 75 % MINO within 90 min at 35 mM glucose, versus 25 % at 5 mM. Released MINO directly kills periodontal pathogens and disrupts biofilms. Additionally, CMGCZ scavenges glucose, inhibits high-glucose-induced macrophage pyroptosis, and reduces senescence-associated protein expression such as p16, p21, β-actin, thereby alleviating gingival fibroblast senescence and reversing the inflammatory microenvironment (Fig. 3B) (Fig. 3C). Emerging GOx-based strategies have been developed for periodontal therapy. For instance, Wang et al. combined Au NPs with GOx to cascade-catalyze ROS generation for periodontal pathogen eradication. [30] Conjugated GOx oxidizes glucose to H_2_O_2_ and gluconic acid. Au NPs exhibit peroxidase (POD)-like activity, converting H_2_O_2_ to hydroxyl radicals (•OH) to degrade biofilms (Fig. 3D). Similarly, another study utilized Au NPs-MnCO to combat diabetic periodontitis. [34] The Au NPs-glucose cascade generates H_2_O_2_ and •OH, which degrade MnCO to release CO gas in situ (Fig. 3E). Controlled CO release effectively suppresses proinflammatory cytokines (*e.g.* TNF-α, IL-1β, IL-6), synergizing with Au NPs against periodontitis.

Furthermore, PBA-based chemical structural response strategies have been reported for hypoglycemic and anti-inflammatory applications. PBA forms reversible ester bonds with the cis-diol configuration of glucose.Under hyperglycemic conditions, glucose competitively binds to PBA, inducing carrier swelling/disintegration to release drugs. For example, a smart glucose-responsive microneedle patch dynamically releases hypoglycemic hormones and regulates blood glucose in animal models [32]. Under physiological pH conditions, PBA binds to glucose to form negatively charged boronate ester complexes. When blood glucose levels rise, the material matrix becomes negatively charged, accelerating insulin release through electrostatic repulsion(Fig. 3F). Conversely, when blood glucose levels decrease, the material matrix becomes positively charged, repelling the positively charged glucagon and promoting its rapid release(Fig. 3G). Glucose concentration gradients dynamically regulate the release ratio of both hormones to maintain glycemic homeostasis (Fig. 3H). However, such materials remain unexplored in periodontal inflammatory contexts. Furthermore, Feng et al. designed a glucose/ROS dual-responsive hydrogel network for treating chronic periodontitis in diabetic rats. [31] Epigallocatechin gallate (EGCG) and PBA are co-loaded into the hydrogel through reversible boronate ester bonds. Glucose molecules form more stable ester bonds with PBA, thereby competitively displacing EGCG's boronate ester bonds. Under glucose or ROS stimulation, cumulative EGCG release exceeds unstimulated conditions. Released EGCG promotes periodontitis treatment via pleiotropic effects: antioxidant, anti-inflammatory, antibacterial, and osteometabolic regulation.

Glucose-responsive materials offer significant benefits but also present inherent limitations. Although the activity of GOx is maintained during carrier preparation, the attenuation of enzymatic reactions hinders continuous glucose sensing. [35] Additionally, the oral microenvironment contains small molecules such as lactic acid and uric acid, which have similar structures or chemical properties to glucose. [36] These molecules can cause non-specific binding with glucose-sensitive materials, leading to false positives or false negatives.

### 2.3 Enzyme responsive materials

Enzyme-responsive materials are a class of intelligent biomaterials that rely on "enzyme-substrate" molecular recognition mechanisms to achieve structural transformation or functional activation. [37] Their mechanisms of action include controlling drug release via enzyme-cleavable motifs and constructing enzyme-activated prodrug systems. The former strategy involves designing specific enzyme-sensitive functional groups or linkers. Under the action of target enzymes, enzymatic activity induces bond cleavage or structural changes to trigger drug release. [38,39]The latter approach depends on target enzymes to catalyze the conversion of inactive prodrugs into functional therapeutic agents. In periodontitis, abnormally accumulated enzymes provide accessible targets for developing these responsive materials. [40,41] Destructive enzymes, such as gingipains and matrix metalloproteinases (MMPs), degrade collagen and other extracellular matrix (ECM) components in periodontal tissues. These effects lead to the destruction of periodontal support structures and the exacerbation of inflammatory responses. [[42], [43], [44], [45]] Regenerative enzymes (alkaline phosphatase (ALP)/TIMPs) maintain repair potential by promoting ECM remodeling and bone regeneration. [46,47] The dynamic balance between these two enzyme categories regulates disease progression.

**Fig. 4:**
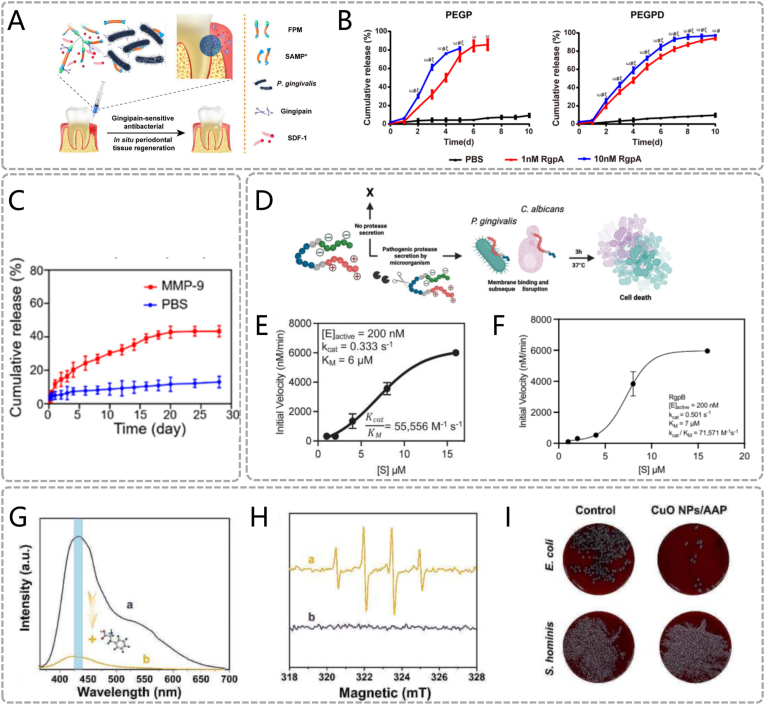
Enzyme responsive materials. (A) Schematic diagram of the enzyme-responsive mechanism of PEGPD@SDF-1. (B) The release profiles of SAMP from the hydrogels PEGP and PEGPD over a 10 - day period in various solutions. (A, B) Reproduced with permission. [38] Copyright 2021, American Chemical Society. (C) In vitro, the release rate patterns of CuTA NSs from the TM/BHT/CuTA hydrogel within PBS at 37 °C, with and without MMP - 9. Reproduced with permission. [39] Copyright 2023, American Chemical Society. (D) Descriptive schematic of activatable therapy. The Michaelis - Menten master curve used to describe the reaction rates of the enzyme - substrate systems of (E) SAP9 and (F) RgpB as the substrate concentration increased. (D, E, F) Reproduced with permission. [40] Copyright 2024, The Author(s). (G) Photoemission spectral data of (a) TA-CuO NP-AAP-ALP and (b) TA-CuO NP-AAP-ALP following the incorporation of L-phenylalanine (ALP inhibitor) into the CuO NP-AAP-ALP group. (H) The electron spin resonance (ESR) spectra of CuO NP-AAP-ALP in (a) and CuO NP-AAP in (b) were obtained in a phosphate buffer (20 mM, PH = 7.4). The signal representing DMPO-OH is not observed in the (b) group. (I) Images of colonies with different ALP expression properties under various treatment conditions. [41] Copyright 2024, The Author(s).

Currently, several key enzymes have been identified as critical biomarkers for periodontitis, enabling stimuli-responsive materials to achieve controlled drug release. For instance, Liu et al. designed a gingipain-responsive hydrogel FPM for antibacterial periodontal therapy. FPM consists of anchor peptide-short antimicrobial peptide (SAMP)-anchor peptide. [38] When exposed to gingipain protease, the anchor peptides in FPM are cleaved, releasing the intermediate SAMP (Fig. 4A). In vitro experiments demonstrate that the cumulative release rate of SAMP increases with enzyme concentration in enzyme-containing buffers. In enzyme-free environments, SAMP release is negligible, confirming the enzyme-specific responsiveness of SAMP release (Fig. 4B). Additionally, an MMP-responsive composite TM/BHT/CuTA treats periodontitis through antibacterial and ROS-scavenging functions. [39] The TM/BHT hydrogel encapsulates CuTA nanozymes via ester bonds. Elevated MMP-9 in periodontitis specifically hydrolyzes these ester bonds, triggering gel degradation and CuTA release. In MMP-9-containing buffers, cumulative CuTA release (40 %) significantly exceeds enzyme-free conditions (<15 %), validating on-demand release (Fig. 4C). CuTA mimics SOD-like and CAT-like enzymatic activities, exhibiting broad-spectrum antibacterial properties and ROS scavenging capacity.

Moreover, enzyme-activated prodrug systems have been reported for antibacterial bioengineering applications. For instance, a protease-activatable prodrug achieves controlled antimicrobial peptide (AMP) release via proteases overexpressed by Candida albicans and *Porphyromonas gingivalis (P. gingivalis).* [40] The prodrug system comprises three components: AMP, quencher, and protease-specific cleavage sites (Fig. 4D). The negatively charged quencher neutralizes AMP's positive charge through electrostatic interactions, rendering the prodrug inactive until activation. Only when pathogen-derived proteases cleave specific sites in the prodrug, the quencher separates from the AMP, and the AMP regains antibacterial activity. RgpB (protease from *P. gingivalis*) exhibits higher catalytic efficiency than SAP9 (protease from *C. albicans*), enabling faster AMP release in infected microenvironments (Fig. 4E) (Fig. 4F). Zhuang et al. developed an ALP-activated prodrug system (CuO NPs/AAP) for on-demand sterilization. [41] Pathogen-derived ALP catalyzes dephosphorylation of AAP (sodium 2-phospho-L-ascorbate) to generate active ascorbic acid (AA) (Fig. 4G). CuO NPs exhibit ascorbate oxidase- and peroxidase-like activities, converting AA into ROS (Fig. 4H) (Fig. 4I). In vitro antibacterial assays show 90 % eradication efficiency against high-ALP-expressing *E. coli* but negligible activity (<5 %) against low-ALP *Staphylococcus.* Although not yet validated in periodontal disease models, the system holds therapeutic potential given ALP overexpression in inflammatory periodontal microenvironments.

Despite these encouraging advancements, enzyme-responsive biomaterials face inherent limitations. Variability in enzyme expression among individuals may pose challenges to the specificity required for targeted therapies. [42] Secondly, these materials face challenges in physical stability and storage during clinical transportation. Enzyme-sensitive groups (*e.g.* ester bonds) are prone to non-specific hydrolysis under high-temperature and high-humidity conditions, compromising controlled drug release performance. [48] Additionally, freeze-thaw cycles of nanocarriers may induce conformational changes in enzyme-responsive sites, leading to reduced enzymatic cleavage efficiency. [49]

### 2.4 Redox responsive materials

Redox-responsive materials are functional systems that exhibit microenvironment-specific responses via bond cleavage or structural changes under oxidative/reductive conditions. Their design leverages pathological gradients of ROS and glutathione (GSH). Periodontitis progression is characterized by dynamic oxidative stress evolution. Dental plaque biofilms stimulate neutrophil respiratory bursts to overproduce ROS, upregulating inflammatory cytokines (*e.g.* TNF-α, IL-1β) and forming a positive feedback loop between inflammation and oxidative stress. [50] Excessive ROS exacerbates bone resorption by activating osteoclast differentiation while suppressing osteogenic functions of periodontal ligament stem cells, disrupting bone remodeling homeostasis. [51] Simultaneously, the endogenous antioxidant GSH accumulates in response to elevated ROS levels, protecting cells through alleviation of oxidative stress. [52] However, chronic exposure to high ROS concentrations induces mitochondrial dysfunction and GSH reductase (GR) activity suppression, causing GSH depletion. [53] Thus, redox-responsive designs must dynamically adapt to disease stages.

**Fig. 5:**
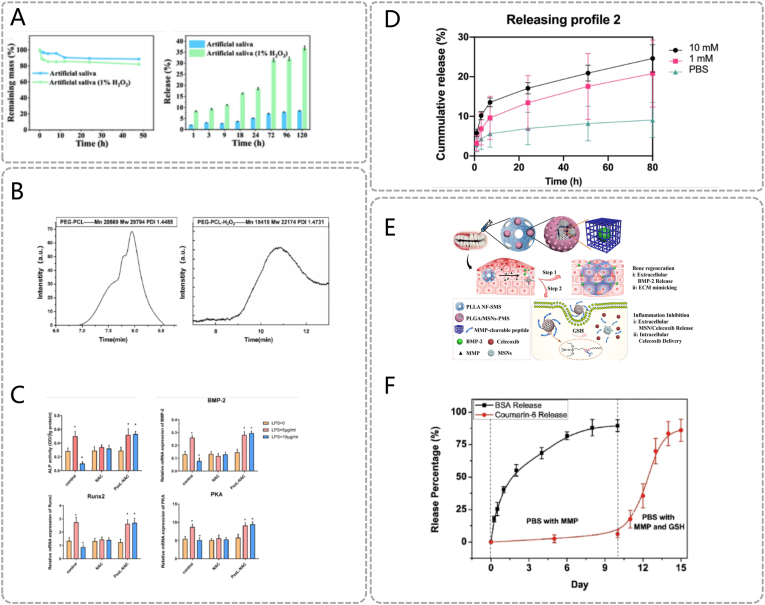
Redox responsive materials. (A) The remaining mass and drug release profile of MNs in artificial saliva with or without H_2_O_2_ under 37 °C. Reproduced with permission. [54] Copyright 2023, The Authors. (B) Gel permeation chromatography of PEG-ss-PCL treated with or without ROS. (C) Osteogenic differentiation of hPDLSCs treated with different concentrations of LPS (0, 5, 10 μg/mL) in the absence or presence of NAC and PssL-NAC. Schematic diagrams of the obtained ALP activity, BMP-2 mRNA expression, Runx2 mRNA expression and PKA mRNA expression. (B, C) Reproduced with permission. [55] Copyright 2021, Elsevier. (D) The release patterns of RB with a loading efficiency of 5.2 % in PBS solutions featuring various GSH concentrations (0, 1, and 10 mM). Reproduced with permission. [55] Copyright 2022, Elsevier. (E) PLGA/MSNs-PMS immobilized PLLA spongy nanofibrous micro - scaffold and the related dual - response mechanism. (F) Release kinetics of the micro-scaffold carrying rhodamine-labeled BMP-2 (using fluorescently labeled BSA as a model) and CEL (using coumarin-6 as a model) complex with a dual-dependence on matrix metallo proteinases (MMP) and GSH. (E, F) Reproduced with permission. [58] Copyright 2021, Elsevier.

In recent years, redox-sensitive materials—those containing reducible/oxidizable bonds such as disulfide bonds, boronate esters, thioether bonds, B-N coordination, guanidinium groups, manganese oxide particles, and MPNs—have been explored for binding and delivering therapeutic payloads in periodontal treatment. For instance, Song et al. developed a thioketal-based microneedle system (poly(lactic-co-glycolic acid) (PLGA)-thioketal (TK)-poly(ethylene glycol) (PEG)) mimicking bee stings for periodontitis therapy. [54] Under high ROS conditions in periodontal lesions, TK bonds undergo oxidative cleavage, causing microneedle tip disintegration and metronidazole (MZ) release. Compared to artificial saliva alone, H_2_O_2_-containing artificial saliva significantly enhanced drug release (Fig. 5A). Released MZ potently inhibited biofilm formation by periodontal pathogens (*P. gingivalis* and *F. nucleatum*) and reduced bacterial colonization. Additionally, Qiu et al. encapsulated N-acetylcysteine (NAC), a ROS scavenger, within thioether-based NPs to remodel the inflammatory periodontal microenvironment and promote osteogenesis. [55] In inflammatory conditions, ROS disrupt thioether bonds to degrade the polymer matrix and release NAC (Fig. 5B). ROS not only act as stimuli to activate responsive materials but also serve as therapeutic targets for intervention. NAC-mediated ROS scavenging effectively upregulated ALP activity in human periodontal ligament stem cells (hPDLSCs) and enhanced expression of BMP-2, core-binding factor α2 (Runx2), and protein kinase A (PKA), thereby promoting osteogenic differentiation and bone formation (Fig. 5C). Similarly, other studies demonstrate that ROS-responsive/ROS-scavenging material designs can effectively mitigate periodontal inflammation and enhance soft/hard tissue regeneration. [21,56]

On the other hand, recent studies have also focused on designing redox-responsive materials targeting GSH overaccumulation in periodontitis. For instance, a nanoparticle system (RB) achieves drug-controlled release via a GSH-sensitive poly disulfide cell-penetrating poly(disulfide) (CPD) layer. [57] GSH acts as a reductant to break disulfide bonds in the CPD layer through thiol-disulfide exchange reactions, releasing baicalein (BAI). In vitro GSH-responsive release assays revealed that RB released only approximately 20.8 % of BAI in 1 mM GSH-PBS over 80 h, increasing to 24.6 % under 10 mM GSH (Fig. 5D). BAI, an NF-κB signaling pathway inhibitor, effectively downregulates proinflammatory cytokines such as IL-6 and IL-8. Similarly, a porous PLGA microsphere system (PLGA/mesoporous silica (MSN)-PMS) enables MMP/GSH dual-responsive on-demand drug delivery in inflammatory microenvironments. [58] MSN clusters are conjugated to PLGA shells via MMP-cleavable peptides. Upon MMP exposure, the PLGA microspheres undergo specific cleavage, resulting in the release of BMP-2 and Celecoxib (CEL)-loaded MSNs. BMP-2 directly stimulates ECM remodeling to promote bone regeneration. MSNs form clustered structures (MSN-SH) via surface thiol (-SH) functionalization. Following cellular internalization, intracellular GSH reduces disulfide bonds (-S-S-), disassembling clusters and releasing CEL (Fig. 5E). In simulated conditions, MSN clusters release approximately 80 % of payload within five days (Fig. 5F). CEL, a selective cyclooxygenase-2 (COX-2) inhibitor, suppresses periodontal inflammation. [59] While GSH-responsive materials offer novel therapeutic options, clinicians must account for potential GSH depletion in chronic periodontitis patients, which may impede drug release efficacy. Co-administration with mitochondrial-targeted antioxidants is recommended to enhance therapeutic outcomes.

Although redox-responsive biomaterials show promise, they currently face several limitations. Primarily, high concentrations of ROS and GSH under acute inflammatory conditions can trigger rapid drug release from responsive carriers, potentially diminishing their clinical efficacy. [60] Additionally, the heterogeneity of the redox environment across different individuals and disease states poses a challenge to achieving consistent and predictable responses from these materials. [61] For example, the oxidative microenvironment in high-risk populations (*e.g*. smokers, diabetics) may exceed the response thresholds of conventional materials, leading to drug burst release or premature carrier degradation. [51,62] Based on the above discussion, developing redox-responsive materials requires establishing a multistage optimization framework. Firstly, material selection and drug-loading strategies should dynamically adapt to disease staging. Secondly, personalized delivery systems should be designed through risk stratification to target individuals with high oxidative stress. Additionally, real-time biosensing technologies must be integrated to enable dynamic modulation of therapeutic regimens.

### 2.5 Endogenous electricity responsive materials

In periodontal tissues, endogenous electrical signals trigger cell membrane potential changes that drive the fluxes of Ca^2+^, Na^+^, K^+^, and Cl^−^, thereby modulating intracellular signaling pathways. Activation of these downstream signaling cascades ultimately promotes the proliferation and differentiation of fibroblasts, osteoblasts, and periodontal ligament cells. [17,63] Furthermore, these electrical signals can guide the directed migration of cells involved in periodontal regeneration to the injury site, accelerating the healing process. [64,65] In most tissue engineering applications, the intrinsic conductive networks of materials are sufficient to achieve functional modulation. In scenarios requiring precise spatiotemporal control or enhanced therapeutic outcomes, synergistic application of exogenous electric fields can amplify biological effects.

**Fig. 6:**
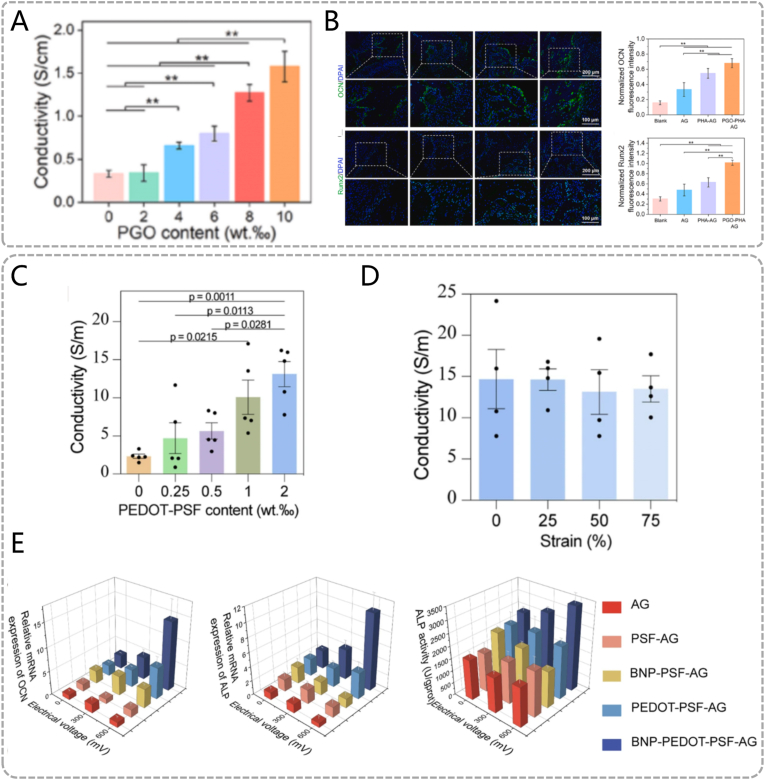
Endogenous electricity responsive materials. (A) Conductance properties of scaffolds with diverse contents of PGO. (B)Immunofluorescence staining of osteocalcin (OCN) and Runx2 (green) in defect areas, and normalized fluorescence intensity. (A, B) Reproduced with permission. [66] Copyright 2022, The Author(s). (C) Electrical conductivities of hydrogels containing varying amounts of PEDOT – PSF. (D) Conductivity of the BNP-PEDOT-PSF-AG hydrogel following compression. (E) Osteogenesis-related gene expressions of OCN (a) and ALP (b), as well as the ALP activity, of PDLSCs on various hydrogels under different ES potentials. (C, D, E) Reproduced with permission. [67] Copyright 2024, The Author(s). (For interpretation of the references to colour in this figure legend, the reader is referred to the Web version of this article.)

Conductive polymers such as polydopamine (PDA), poly(3,4-ethylenedioxythiophene) (PEDOT), and polypyrrole (PPy) exhibit conductivity comparable to metals and inorganic semiconductors, coupled with excellent biocompatibility and ease of synthesis, making them widely applicable in periodontal tissue engineering. For instance, Li et al. developed a PDA-mediated graphene oxide (GO)-based conductive scaffold to enhance diabetic periodontal bone regeneration. [66] While pristine GO has poor conductivity, PDA functionalization significantly enhances its conductivity (Fig. 6A). PDA stabilizes polydopamine-reduced grapheane oxide (PGO) dispersion within the scaffold via π-π interactions and hydrogen bonding, preventing aggregation and maintaining conductive pathways. The scaffold transmits endogenous electrical signals to BMSCs by homogenizing local electric fields, thereby promoting Ca^2+^ influx. This activates intracellular signaling cascades that upregulate osteogenic genes such as *OCN* and *Runx2* (Fig. 6B). Furthermore, PEDOT-mediated hydrogels have been shown to promote periodontal tissue regeneration by transmitting endogenous bioelectrical signals. [67] The π-π conjugated structure of PEDOT enables electron transport along polymer chains via hopping or delocalization mechanisms. [68] PEDOT also maintains electrical stability under mechanical stress and long-term implantation (Fig. 6C) (Fig. 6D). Similar to PDA systems, conductive polymers enhance Ca^2+^ influx in PDLSCs through inherent conductivity. Exogenous electrical stimulation can amplify these biological effects. In vitro osteogenesis assays demonstrate that increasing external potentials strengthens the material's ability to upregulate osteogenic genes (*OCN, ALP*) and ALP activity (Fig. 6E).Similarly, Qin et al. developed a PPy-based conductive hydrogel that responds to endogenous electric fields to promote periodontal regeneration and coordinate osseoperception. [69] The transmitted endogenous electric field enhances neurite growth and the release of neuropeptides by increasing intracellular Ca^2+^ concentration, improving osseoperception and osseointegration around dental implants. However, this material has yet to be explored in the context of periodontal inflammation.

The utilization of electroactive materials to boost endogenous bioelectrical signals in periodontal therapy shows considerable promise, yet it also faces several obstacles. Primarily, the intricate microenvironment of the oral cavity, characterized by variations in pH, temperature, and humidity, may pose challenges to the stable electrical conductivity of these materials. [70,71] Additionally, the diverse needs of different cells and tissues for electrical signals mean that finely tuning the conductivity of electroactive materials to deliver suitable signal strengths continues to be a significant hurdle. [72,73]

## 3 Exogenous stimuli to regulate periodontal disease

Despite the spatial targeting advantages provided by physiological stimuli for periodontal therapy, limitations exist in achieving precise temporal control. Specifically, upon reaching the inflamed site, endogenous stimuli can immediately trigger the release of therapeutic agents, potentially leading to issues such as short treatment duration or excessively high local drug concentrations. Exogenous-responsive biomaterial platforms respond to a variety of external stimuli, including light, thermals, magnetic fields, vibration, and ultrasound. In contrast, these systems enable on-demand delivery of therapeutics with instantaneous controllability, enhancing both specificity and precision of treatment.

### 3.1 Light responsive materials

Light-responsive materials include photothermal agents, photosensitizers, and photocatalysts. Photothermal materials absorb light to trigger localized surface plasmon resonance (LSPR) or molecular vibration relaxation. They efficiently convert light energy into heat. [74] Photosensitizers, under specific wavelength light irradiation, can produce ROS. This occurs through electron transfer interactions with surrounding oxygen molecules. [75] Photocatalytic materials focus on the separation and migration of photogenerated carriers. Through band structure design, they promote electron-hole pairs to participate in redox reactions. [76] Among all response signals, optical signals have unique advantages such as high sensitivity, fast response speed, high spatial resolution and modulatable as needed. [77,78] Given these advantages, various photoresponsive materials have demonstrated significant potential for applications in periodontal therapy.

**Fig. 7:**
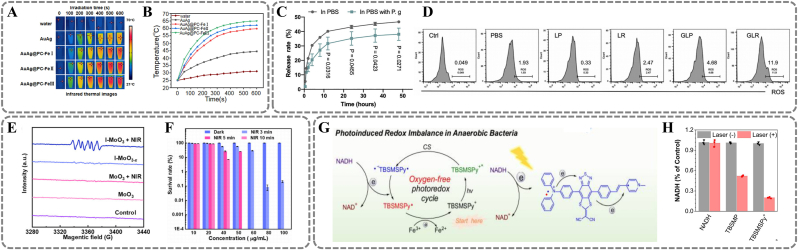
Light responsive materials. (A) Thermal images of AuAg, AuAg@PC-FeI, AuAg@PC-FeII, and AuAg@PC-FeIII solution (100 μg/mL) under irradiation for 808 nm 10 min at 2.5 W/cm2. (B) temperature profile. (A, B) Reproduced with permission. [80] Copyright 2022, The Authors. (C) Release of gallium porphyrin from GLR in PBS or the *P. gingivalis* supernatant. (D) Flow analysis of intracellular ROS produced in *P. gingivalis* after treatment with PBS, LP (liposome), LR (liposome-containing red blood cell membranes), GLP (liposome loaded with gallium porphyrins), or GLR (liposome-containing red blood cell membranes loaded with gallium porphyrins) under blue light. (C, D) Reproduced with permission. [85] Copyright 2024, American Chemical Society. (E) Electron spin resonance spectra of ·O2− trapped by DMPO in the presence of MoO3 or I-MoO3−x under dark or irradiation of NIR laser (808 nm, 1 W/cm2). DMPO, 5,5-dimethyl-1-pyrroline-N-oxide; DPBF, 1,3-diphenylisobenzofuran. NIR, near-infrared. (F) Survival rate of *S. aureus*. (E, F) Reproduced with permission. [86] Copyright 2023, The Authors. (G) Schematic illustration of photoinduced redox imbalance in anaerobic bacteria through multi-step electron transfer of TBSMSPy^+^. (H) Quantitative analysis of NADH level in bacterial suspension treated with TBSMP and TBSMSPy^+^ at laser irradiation and without irradiation. (G, H) Reproduced with permission. [88] Copyright 2024, The Authors. (For interpretation of the references to colour in this figure legend, the reader is referred to the Web version of this article.)

Phototherapy, based on its antibacterial and anti-inflammatory mechanisms, is primarily categorized into photothermal therapy (PTT) and photodynamic therapy (PDT). PTT leverages near-infrared (NIR) light to elevate local temperatures above 50 °C, thereby compromising cellular membranes and inducing protein denaturation, effectively eliminating bacteria. [79] For instance, a MPN-coated branched AuAg nanoparticle (AuAg@PC-Fe) treats periodontitis via photothermal antimicrobial action. AuAg NPs exhibit LSPR, converting NIR light to heat. The MPN coating (PC-Fe) enhances this efficiency, raising temperatures to 50 °C under laser irradiation (Fig. 7A) (Fig. 7B). [80] However, the risk of damaging adjacent healthy tissues with excessive heat or insufficient antibacterial effects with suboptimal temperatures presents a conundrum when employing PTT alone. [81] Consequently, integrating PTT with functional agents represents a promising approach to enhancing antibacterial efficacy without adversely affecting surrounding normal tissues. For example, sodium nitroprusside-loaded Prussian blue nanozyme (SPBzyme) shows notable antioxidant and photothermal properties. [82] SPBzyme raises temperatures to about 45 °C within 10–15 min, demonstrating mild and efficient photothermal performance. This effect arises from absorbed light energy being converted to heat through non-radiative relaxation. Moreover, in periodontal inflammation, overexpressed H_2_O_2_ triggers the redox decomposition of nitroprusside, releasing NO for anti-inflammatory and antibacterial effects. Notably, NIR light accelerates NO release, enabling on-demand controlled release and disease modulation under photothermal activation.

PDT involves light-excited photosensitizers rapidly generating ROS. This triggers irreversible cytotoxic effects like lipid peroxidation, protein denaturation, and DNA damage. [83] However, the tightly structured outer membrane of Gram-negative bacteria such as *P. gingivalis* often impedes photosensitizer penetration, posing challenges for PDT application. [84] To address this, Tang et al. developed red blood cell membrane-mimicking nanovesicles. These enhance photosensitizer targeting based on *P. gingivalis*'s iron uptake mechanism. [85] *P. gingivalis* relies on red blood cell aggregation and lysis to acquire iron and heme. Gallium porphyrin-loaded nanocarriers precisely target and adhere to *P. gingivalis*. The bacteria then lyse the carriers, ingesting gallium porphyrin (Fig. 7C). Internalized gallium porphyrin replaces iron porphyrin, causing metabolic disruption. Under blue light excitation, deposited porphyrin molecules transfer energy to oxygen via electron transitions. This generates ROS like singlet oxygen (^1^O_2_), killing *P. gingivalis* (Fig. 7D). Additionally, as an oxygen-dependent therapy, traditional PDT faces limitations when addressing hypoxic regions within biofilms. Li et al. developed intercalated MoO_3_ nanobelts (I-MoO_3_-x) for periodontitis treatment in hypoxic environments. [86] Sodium ions and water intercalation narrow I-MoO_3_-x's bandgap, enhancing NIR absorption and electron excitation. Intercalation also creates oxygen vacancies, suppressing electron-hole recombination and accelerating electron transfer. These electrons react with water/residual oxygen to generate superoxide radicals (·O_2_^−^), peroxides (O_2_^2−^), and hydroxyl radicals (·OH). Unlike traditional PDT, this process does not rely heavily on molecular oxygen to produce singlet oxygen (^1^O_2_) (Fig. 7E). ROS regenerates oxygen through disproportionation/Haber-Weiss/Fenton reactions, forming an "oxygen cycle" to reduce oxygen demand. In vitro experiments show I-MoO_3_-x exhibits excellent NIR photodynamic antibacterial activity. Its efficacy increases with material concentration and irradiation time (Fig. 7F).

Unlike photosensitizers, photocatalytic materials are reusable and can couple with photothermal effects for long-term antibacterial action. [76] Kong et al. reported a novel Bi_2_S_3_/Cu-TCPP nanocomposite. It aims to treat periodontitis via enhanced photocatalysis and photothermal effects. [87] Bi_2_S_3_ and Cu-TCPP are tightly integrated through heterojunction interfaces, forming a Z-scheme band structure. This Z-type heterojunction facilitates electron transfer from Bi_2_S_3_'s conduction band (CB) to Cu-TCPP's valence band (VB). It suppresses electron-hole recombination, enhancing ROS production. Additionally, PTT based on Bi_2_S_3_ NPs promotes Cu^2+^ ion release, synergistically eradicating dense biofilms. Furthermore, a new photocatalyst, TBSMSPy^+^, induces photoinduced redox imbalance (PIRI) for treating anaerobic bacterial periodontitis. [88] Under light irradiation, TBSMSPy^+^ catalyzes NADH oxidation to NAD^+^ via intramolecular multi-step electron transfer (Fig. 7G). Light excites electron transfer from TPA to the pyridinium cation, forming a TPA^+^-BSM^-^-Py^+^ charge-separated state. In anaerobic conditions, the pyridinium cation captures electrons to form Py·, while TPA oxidizes NADH to NAD^+^. Py· transfers electrons to Fe^3+^ (a bacterial metabolite), resetting the system to its initial state, completing the catalytic cycle. Under anaerobic conditions, TBSMSPy^+^'s NADH oxidation turnover frequency is 60.7 min^−1^, much higher than similar catalysts. After TBSMSPy^+^ treatment, anaerobic bacteria, such as *P. gingivalis*, show an 80 % reduction in NADH content (Fig. 7H). In vitro experiments show that reduced NADH levels disrupt ATP synthesis and DNA replication in *P. gingivalis*, achieving a 99.2 % kill rate.

Despite significant advancements in the application of photoreactive materials, several critical challenges remain to be addressed. One primary hurdle is the limited efficacy of photobiomaterials in deep tissue regions or areas that are difficult for light to reach. [78] Additionally, the spatial targeting of light-controlled materials is insufficient, which could lead to potential off-target effects. [89]

### 3.2 Thermal responsive materials

Thermosensitive materials exhibit unique application value in periodontal therapy due to their temperature-dependent phase transition mechanism. At room temperature, these materials remain in a liquid (sol state), facilitating easy application or injection into the affected area. [90] This non-invasive administration method significantly enhances patient compliance and treatment experience. Upon entering the higher temperature oral environment, the polymer chains undergo contraction and aggregation, rapidly transforming the material into a gel state. [91] This phase transition allows for the formation of a stable drug reservoir at the lesion site, extending the release duration and ensuring sustained, effective action of the medication on the affected area. Moreover, the gel state enhances the tissue adhesion of material, reducing drug leakage and increasing local drug concentration.

**Fig. 8:**
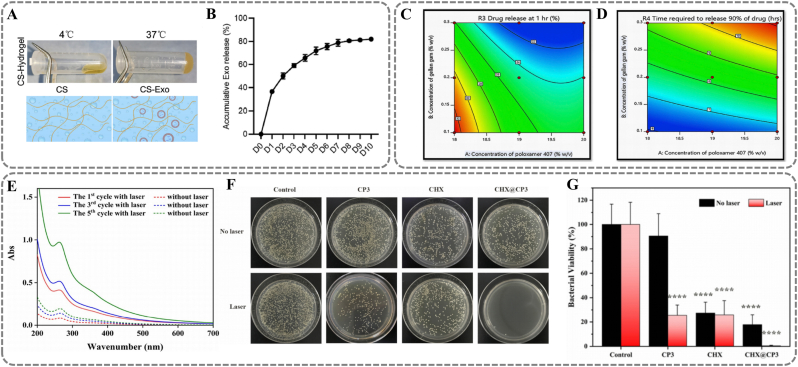
Thermal responsive materials. (A) Synesis process and schematic illustration of CS hydrogel. (B) The release curves of DPSC-Exo from the CS hydrogel. (A, B) Reproduced with permission. [92] Copyright 2020, The Authors. (C) Contour plots for drug release at 1hr. (D) Contour plots for time required to release 90 % of drug. (C, D) Reproduced with permission. [94] Copyright 2019, Craniofacial Research Foundation. (E) Ultra violet (UV) absorbance of triggered release. (F) The control and CHX@CP3 groups with and without NIR irradiation of *S. aureus*. (G) The bacterial viability of the control and CHX@CP3 with and without NIR irradiation of *S. aureus*. (E, F, G) Reproduced with permission. [95] Copyright 2020, Elsevier. (For interpretation of the references to colour in this figure legend, the reader is referred to the Web version of this article.)

Currently, various thermoresponsive material matrices, such as CS, curdlan, cellulose ether derivatives, and triblock copolymer systems, have been extensively explored for their value in the targeted delivery of drugs for treating periodontal disease. For example, a chitosan hydrogel incorporating dental pulp stem cell-derived exosomes (DPSC-Exo/CS) was developed to alleviate periodontitis in mice. [92] β-GP serves as a crosslinker to regulate structural phase transitions of the material. [93] At 4 °C, the phosphate groups of β-GP partially neutralize the positive charges of CS amino groups. This weakens intermolecular electrostatic repulsion, maintaining a sol state. At 37 °C, β-GP promotes hydrogen bonding and hydrophobic interactions between CS chains, driving gel network crosslinking (Fig. 8A). The gelled CS adheres to periodontal tissues and gradually releases DPSC-Exo. In vitro release experiments show that about 80 % of DPSC-Exo is released within 7 days, with sustained release over 10 days, enabling long-term efficacy (Fig. 8B). DPSC-Exo regulates macrophage polarization via miR-1246. It suppresses pro-inflammatory phenotypes (M1) and promotes anti-inflammatory phenotypes (M2), alleviating periodontal inflammation and tissue damage. Similarly, a dual-responsive hydrogel based on Poloxamer 407 (PEO-PPO-PEO triblock copolymer) and gellan gum delivers moxifloxacin (MFX). [94] Gellan gum enhances mechanical strength through cationic crosslinking, while Poloxamer prolongs drug release via temperature-triggered micelle crosslinking. At low temperatures, Polyethylene oxide (PEO) chains of Poloxamer 407 form hydrogen bonds with water molecules. Poly(propylene oxide) (PPO) chains are surrounded by hydrated layers, preventing micelle formation. Upon heating, hydrogen bonds break, and dehydrated PPO aggregates hydrophobically to form micelle cores. Additionally, the Poloxamer micelle network interpenetrates with the ion-crosslinked gellan gum network, forming a denser barrier to slow MFX diffusion. In vitro experiments show that increasing Poloxamer 407 and gellan gum concentrations reduces MFX release rates, achieving long-term controlled release (Fig. 8C) (Fig. 8D). Released MFX interferes with bacterial DNA metabolism by inhibiting DNA topoisomerase II/IV activity. Additionally, Tong et al. developed a thermosensitive micelle system. It uses chlorhexidine acetate (CHX) as a carrier and curdlan and PDA as the matrix for treating periodontitis. [95] Chlorhexidine is a broad-spectrum antibacterial agent. It mainly works by disrupting microbial cell membrane integrity. [96] Unlike other materials that undergo thermogelation in the oral microenvironment, PDA-mediated photothermal effects enable more precise control of micelle contraction/expansion and drug release. Under NIR irradiation, PDA's photothermal effect raises the local temperature. This causes partial dissociation of curdlan's triple-helix structure, accelerating drug diffusion (Fig. 8E). After stopping irradiation, the system cools down, and the triple-helix structure reforms, causing the micelle network to contract and form a sustained-release barrier. In vitro experiments achieved on-demand stepwise CHX release through three intermittent NIR irradiations. Due to the synergy between photothermal effects and antibacterial drugs, the system achieves a 99.53 % antibacterial rate against periodontal pathogens (Fig. 8F) (Fig. 8G).

Thermoresponsive materials demonstrate significant potential in periodontal treatment, yet they face numerous challenges in practical application. Firstly, the presence of ions in the oral microenvironment can affect the solvation degree of the materials, altering their thermoresponsive behavior. [97] Secondly, research has revealed that the local temperature within the periodontal microenvironment varies according to anatomical location. This necessitates the design of thermosensitive materials to function within specific temperature ranges, thereby increasing the technical and financial complexity of material design. [98]

### 3.3 Magnetism responsive materials

Magnetic responsive materials exhibit unique advantages in periodontal treatment. Magnetic-responsive materials refer to functional materials that produce physical responses under an applied magnetic field. Their core components often include magnetic elements such as iron, cobalt, nickel, or their oxides (*e.g.* Fe_3_O_4_, iron oxide NPs). [99] Magnetic fields possess high penetrability and are not affected by the absorption and scattering of biological tissues, allowing precise guidance of magnetic NPs to the lesion site. [100] Moreover, the nonlinear response characteristics of magnetic NPs enable localized high-concentration drug accumulation and release at specific magnetic field strengths, thereby enhancing therapeutic efficacy and reducing systemic side effects. [101]

**Fig. 9:**
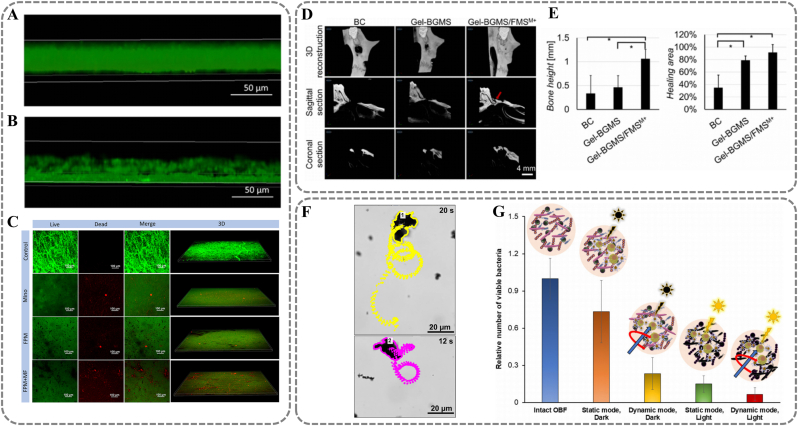
Magnetism responsive materials. (A, B) Penetrating effect of FPM on biofilms. Transverse cross-sectional CLSM images of periodontal biofilms treated with FPM before and after magnetic motivation. (C) Representative live/dead and 3D reconstruction images of *P. gingivalis* on HA disks (dead bacteria stained red; live bacteria stained green). (A, B, C) Reproduced with permission. [102] Copyright 2023, The Author(s). (D) Images of 3D reconstruction and sectional view of the effects of FMSs on bone defect healing at 3 months post-surgery. (E) The quantitative analyses of the regenerated bone height along the mesial surface of M1 and the bone healing area percentage. (D, E) Reproduced with permission. [104] Copyright 2023, The Authors. (F) Time-lapse microscopy images illustrating tracking lines of swimming of Fe_3_O_4_@PEI/BiVO_4_ magnetic microrobots under a transversal rotating magnetic field using the predefined clockwise circular propulsion mode. (G) Antibiofilm activity of Fe_3_O_4_@PEI/BiVO_4_ photoactive magnetic microrobots under different experimental conditions. (F, G) Reproduced with permission. [105] Copyright 2022, American Chemical Society. (For interpretation of the references to colour in this figure legend, the reader is referred to the Web version of this article.)

Based on these properties, Fe_2_O_3_, Fe_3_O_4_, and Fe-Pt have been extensively studied as platforms for magnetically driven targeting and drug delivery. For instance, MINO-loaded Fe_3_O_4_ NPs can effectively eradicate periodontal biofilms in rat models. [102] MINO inhibits bacterial protein synthesis by binding to the ribosomal 30S subunit. [103] The complex structure of narrow and deep periodontal pockets poses a challenge for the penetration of traditional nanoparticle drug delivery systems. By applying a magnetic field (MF), FPM NPs can be physically driven to penetrate the dental plaque biofilm, enhancing the permeability of the drug in deep tissues (Fig. 9A) (Fig. 9B). Live/dead staining shows that the FPM + MF group significantly increases antibacterial rate compared to FPM NPs group (Fig. 9C). In animal models, rats treated with MINO and FPM showed reduced gingival bleeding. The FPM + MF group had the best outcomes, with pink, firm, elastic gums without bleeding upon probing. Similarly, Zhou et al. developed IR780-loaded Fe_2_O_3_ NPs (FMS) for precise antibacterial effects. [104] Fe_2_O_3_'s superparamagnetism allows it to be driven by a magnetic field to target areas like tooth cervix, penetrating biofilms to deliver IR780. NIR experiments show IR780 has sensitive, recyclable photothermal performance, killing *P. gingivalis* by releasing ROS. Mixing FMS with osteogenic hydrogel precursors enables targeted sterilization under NIR via magnetic force, aiding bone defect healing. Micro-CT at three months post-operation shows FMS + MF groups have significantly higher bone levels than other groups, indicating better bone healing (Fig. 9D) (Fig. 9E). Martinez's team developed a magnetically driven light response microrobot Fe_3_O_4_@PEI/BiVO_4_ for the treatment of periimplantitis. [105] With Fe_3_O_4_ as the core material, its movement can be precisely controlled via an external magnetic field. Unlike traditional materials, Fe_3_O_4_@PEI/BiVO_4_ achieves coordinated motion in programmable paths (Fig. 9F). Under magnetic fields, individuals merge into larger entities, improving efficiency and penetration. BiVO_4_ in the material acts as a photocatalyst for ROS production, synergistically removing peri-implant biofilms. *In vitro* tests show Fe_3_O_4_@PEI/BiVO_4_ kills 77 % of bacteria with a magnetic field only, and this rate increased to 93 % with both magnetic field and light irradiation (Fig. 9G).

Despite the numerous advantages of remotely activated magnetic biomaterials, several challenges persist. Primarily, the anisotropic nature of magnetic NPs may lead to off-target delivery and a reduction in treatment efficacy. [106] For example, rod-shaped Fe_3_O_4_ NPs have higher magnetization than spherical ones, but the surface spin disorder may cause unstable magnetic responses. [107] Additionally, non-uniform aggregation of NPs due to shape effects in vivo may lead to off-target energy deposition. This can cause overheating of healthy tissues or insufficient energy at the target site, reducing treatment efficacy. [106] Additionally, the specialized equipment required for generating and maintaining the magnetic field poses technical hurdles in terms of operational safety and practicality. [102,104,105] Some studies use permanent magnets to drive magnetic particles through biofilms. [102,104] However, high-intensity static magnetic fields may cause mechanical damage in surrounding tissues. Moreover, permanent magnets must be physically close to the target area, making precise control of field gradients and penetration depth difficult. In contrast, electromagnetic field generators drive directional particle movement via rotating magnetic fields, and they show high flexibility and programmability. [105] However, they require power supplies and laser sources, leading to high costs and complex operations. In terms of safety, improper frequency or power control of alternating magnetic fields may cause local overheating and tissue damage. [108]

### 3.4 Vibration responsive materials

Piezoelectric materials are functional materials that convert mechanical stress into electrical signals. [109] Under external force, the internal lattice of piezoelectric materials deforms. This causes relative displacement of positive and negative charge centers, forming a macroscopic polarization field. [109] This polarization effect can induce the release of electrons or hole carriers on the material surface. It further catalyzes redox reactions of substrates or regulates related biological processes. [110,111] Compared with other responsive control systems, vibration-stimulated piezoelectric materials represent a unique advantage in the treatment of periodontal disease. These materials can convert minute mechanical vibrations generated by daily activities, such as chewing, into electrical energy, producing beneficial biological effects without the need for additional energy input. [110] This greatly simplifies the treatment process and reduces costs. Another significant advantage of piezoelectric materials is their ability to enable more precise localized treatment. By directly placing piezoelectric materials at the site of the lesion, they can promote tissue repair and antimicrobial actions spatially specifically, while minimizing impacts on healthy tissues.

**Fig. 10:**
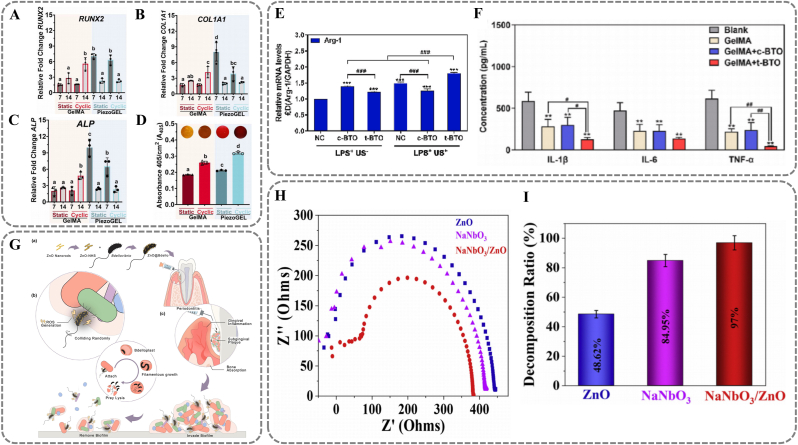
Vibration responsive materials. (A, B, C) Osteogenic differentiation evaluation of GelMA and PiezoGEL hydrogels in vitro, evaluated by the factors including (A) RUNX2, (B) COL1A1, (C) ALP. (D) Normalized absorbance values of Alizarin Red S staining of the formed ECM minerals formed after 14 days of cell culture. (A, B, C, D) Reproduced with permission. [113] Copyright 2023, American Chemical Society. (E) The expression of Arg-1 after treatments. (F) Salivary inflammatory factors (IL-1β, IL-6, and TNF-α) after 12 weeks of post-surgery. (E, F) Reproduced with permission. [114] Copyright 2024, The Authors. (G) The scheme of ZnO@Bdello to eradicate plaque biofilm in periodontitis. Reproduced with permission. [117] Copyright 2022, Elsevier. (H) Electrochemical impedance spectroscopy (EIS) analysis for different prepared samples. (I) Decomposition ratio for the different piezocatalyst. (H, I) Reproduced with permission. [116] Copyright 2022, The Author(s). (For interpretation of the references to colour in this figure legend, the reader is referred to the Web version of this article.)

Considering these benefits, vibration-activated piezoelectric materials, including BaTiO_3_, ZnO, NaNbO_3_ and SrCl_2_, have been extensively studied as platforms for antimicrobial and tissue regeneration. Among them, BaTiO_3_, with high dielectric constants and excellent ferroelectric properties, is most applied in biomedicine. [112] For instance, a BaTiO_3_-based piezoelectric hydrogel (PiezoGEL) achieves non-surgical treatment of periodontitis. [113] PiezoGEL-generated piezoelectric charges inhibit biofilm formation. It does so by inducing ROS production and downregulating adhesion molecules (*e.g.* fimA, porP) of pathogens like *P. gingivalis*. Live/dead staining shows that PiezoGEL kills up to 79 % of bacteria under mechanical stimulation. *In vitro* experiments indicate that PiezoGEL-mediated piezoelectric stimulation can upregulate osteogenesis-related genes (*e.g. RUNX2, COL1A1, ALP*). This promotes osteogenic differentiation and ECM mineralization (Fig. 10A) (Fig. 10B) (Fig. 10C) (Fig. 10D). Liu et al. further explored the mechanisms of in situ regeneration of periodontal inflammatory bone defects mediated by BaTiO_3_-based materials. [114] Piezopotential from mechanically activated hydrogels activates mitochondrial energy metabolism (ATP synthesis). It also modulates macrophage M2 polarization, synergistically promoting bone regeneration. Experimental data show that periodontal ligament stem cells' ATP levels normalize within three days of barium titanate treatment. After five days, osteogenic markers ALP and RunX2 expression significantly increase. Additionally, piezoelectric stimulation markedly elevates the expression of M2 macrophage marker Arg-1. It effectively inhibits pro-inflammatory cytokines IL-1β, IL-6, and TNF-α (Fig. 10E) (Fig. 10F).

In addition to BaTiO_3_, other piezoelectric materials have also garnered attention. ZnO has been reported as an excellent piezocatalyst. It features easy fabrication, low cost, and excellent biosafety. [115,116] Tang et al. utilized ZnO materials in conjunction with bacteriophilic microorganism strategy (ZnO@Bdello) to reduce gingival inflammation. [117] Bdellovibrio bacteriovorus, natural predators of Gram-negative bacteria like *P. gingivalis*, exhibit robust motility. When these microorganisms collide with their prey during feeding, the mechanical pressure activates ZnO nanorods, inducing polarization and the generation of ROS, which enhance the clearance of plaque biofilms (Fig. 10G). *In vitro* experiments show that ZnO@Bdello significantly outperforms ZnO, Bdello, or antibiotics in clearing *P. gingivalis*, *F. nucleatum*, and multispecies biofilms. In vivo experiments confirm ZnO@Bdello's biocompatibility, with no observed liver, kidney damage, or immune abnormalities. Another piezoelectric material, NaNbO_3_, exhibits strong chemical stability and good charge carrier mobility. [118] Sharma and colleagues designed a NaNbO_3_/ZnO binary nanocomposite for cleaning periodontal plaque. [116] Similarly, NaNbO_3_ generates ROS through stress-induced polarization effects to remove dental plaque. Electrochemical impedance spectroscopy (EIS) analysis shows NaNbO_3_ (420.4 Ω) has a smaller charge transfer resistance than ZnO (470.9 Ω), indicating higher electron mobility and catalytic activity (Fig. 10H). Notably, the NaNbO_3_/ZnO binary nanocomposite structure exhibits superior catalytic efficiency under ultrasonic vibration compared to ZnO and NaNbO_3_ alone (Fig. 10I). The degradation rate of target pollutants by NaNbO_3_/ZnO composites is 8.5 times that of ZnO and 1.7 times that of NaNbO_3_.

Despite the promising benefits, vibration-responsive biomaterials face challenges. First, the robustness of piezoelectric materials under varying mechanical stress or environmental conditions is limited. BaTiO_3_ shows high dielectric loss at high frequencies, potentially affecting stability in long-term implantable devices. [119] ZnO may dissolve in acidic body fluid over time. Dissolved free Zn^2+^ could trigger cytotoxic effects. [120] Second, the oxidative stress induced by electrical charges can affect general inflammatory signaling pathways and potentially lead to bacterial mutations that might compromise therapeutic efficacy. [121] Moreover, differences in bacterial surface charge and piezoelectric material polarization direction could influence the selectivity of antimicrobial action. [122]

### 3.5 Ultrasound responsive materials

Ultrasonic response refers to the specific physical or chemical reactions induced in materials under the action of ultrasound. The underlying mechanisms are primarily based on the photothermal effects, mechanical effects, and non-thermal effects (such as acoustic streaming and scattering) triggered by ultrasound waves in a media. [123] As a type of mechanical wave, the high-frequency vibrations of ultrasound can induce cavitation effects within biological tissues. [124] This involves the oscillation, expansion, and collapse of microbubbles in liquid media, generating localized high pressure, high temperature, and micro-jets, which can activate sonosensitizers or facilitate drug release. Additionally, the acoustic radiation force of ultrasound can directionally drive NPs or microbubbles to accumulate in specific regions, enhancing targeting efficiency. [125] Moreover, ultrasound offers advantages such as high safety, strong tissue penetration, non-invasiveness, and excellent spatiotemporal control. [126] Leveraging these properties, platforms utilizing acoustically responsive materials for the treatment of periodontal disease have been extensively researched. Leveraging these properties, platforms utilizing sono-responsive materials for the treatment of periodontal disease have been extensively investigated.

**Fig. 11:**
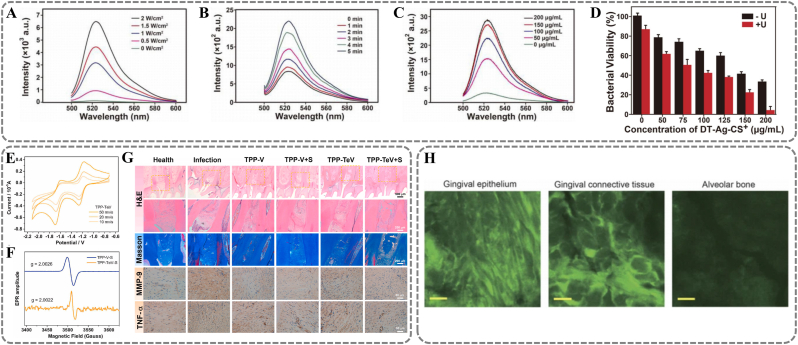
Ultrasound responsive materials. (A, B, C) The fluorescence intensities of DCF in the solutions of DT-Ag-CS upon US irradiation with different power intensities. (D) The bacterial viability rate of *P. gingivalis* after incubation with different concentrations of DT-Ag-CS with or without US irradiation. (A, B, C, D) Reproduced with permission. [128] Copyright 2022, Elsevier. (E) The cyclic voltammogram of TPP-TeV at different scan rates in DMF solution with tetrabutylammonium hexafluorophosphate. (F) EPR spectra of the radical species of TPP-TeV and TPP-V after addition of ultrasonic. (G) H&E, Masson, and the immunohistochemical analysis of MMP-9 and TNF-α staining images of the periodontium after the treatment in decalcified maxilla sections. (E, F, G) Reproduced with permission. [130] Copyright 2023, Elsevier. (H) Magnified views of periodontal tissue in the high-intensity ultrasound-microbubble (HUM-Sc) group. Scale bar = 10 μm. Reproduced with permission. [134] Copyright 2017, The Author(s).

Sonodynamic therapy (SDT), a prominent ultrasonic-responsive anti-inflammatory and antibacterial strategy, employs low-intensity ultrasound irradiation at specific frequencies to activate sonosensitizers, thereby generating reactive ROS for targeted cellular deactivation. [127] For example, a multifunctional nanoplatform DT-Ag-CS^+^ achieves high-efficiency antibacterial effects. [128] It utilizes SDT for periodontitis treatment. Ultrasound irradiation induces periodic pressure oscillations in liquid media, forming cavitation bubbles. Energy released during bubble collapse is captured by mesoporous TiO_2_ (DT). It excites electron-hole pairs, producing ROS. Deposition of Ag enhances TiO_2_'s light absorption through surface plasmon resonance (SPR). It reduces electron-hole pair recombination, increasing ROS quantum yield. Pharmacodynamic analysis shows a significant positive correlation between ROS production and ultrasound power, irradiation time, and DT-Ag-CS^+^ concentration (Fig. 11A) (Fig. 11B) (Fig. 11C). In vitro antibacterial experiments reveal that the minimum inhibitory concentration (MIC) of DT-Ag-CS^+^ against periodontal pathogens is 200 μg/mL (Fig. 11D). This is significantly lower than traditional antibiotics. Traditional SDT relies on oxygen to produce ROS via a Type II mechanism. [129] However, low-oxygen environments in deep periodontal pockets limit its efficacy. To address this limitation, Sun et al. developed a new sonosensitizer, TPP-TeV. [130] It aims to achieve high-efficiency antibacterial effects under hypoxic conditions. Ultrasound cavitation triggers electron transitions in TPP-TeV molecules, creating excited-state intermediates TPP-TeV∗. Through electron transfer processes, these molecules transform into cationic radicals TPP-TeV●. Notably, introducing TeV components lowers TPP-TeV's reduction potential and enhances its electron acceptor capability. (Fig. 11E) (Fig. 11F). TPP-TeV● can react directly with surrounding molecules, such as water molecules or bacterial metabolic intermediates. It generates superoxide anions (O_2_^−^) and hydroxyl radicals (·OH) through an oxygen-independent pathway. In a mouse model of periodontitis, the TPP-TeV combined with ultrasound treatment group showed significant inflammation suppression. This was evident from reduced inflammatory cell infiltration and down-regulated expression of MMP-9 and TNF-α (Fig. 11G).

Beyond SDT, ultrasound technology also demonstrates extensive potential applications in microbubble-mediated drug delivery and piezoelectric material activation. [[131], [132], [133]] For example, a study used ultrasound-microbubble method to transfect NF-κB decoy oligodeoxynucleotides (ODNs) into periodontal tissues. [134] Ultrasound acts on microbubbles, creating transient cavitation effects. It leads to bubble rupture and forms temporary pores in cell membranes. ODNs enter the cytoplasm through these pores efficiently. Detection of ODNs in tissues within 2 h post-ultrasound indicates immediate and efficient release. Ultrasound's focused energy enhances local penetration of microbubbles in gingival tissues. Especially at high intensity, it significantly increases permeability of epithelial and connective tissues (Fig. 11H). Released ODNs inhibit transcription of inflammatory cytokines IL-1β, IL-6, and adhesion molecule ICAM-1. They do so by competitively binding NF-κB proteins. Furthermore, ultrasound acting as an external mechanical stressor can effectively activate piezoelectric materials. Presently, multiple studies have affirmed the potential value of ultrasound-responsive piezoelectric catalytic materials in bacterial eradication and tissue regeneration. [133,135] However, their specific applications in treating periodontal disease remain in the early stages of exploration. [114]

Recent advancements have demonstrated that ultrasonic technology offers growing potential for the functionality of responsive systems; however, there remain challenges to be addressed in the long term. Firstly, the localized thermal effects induced by ultrasound may lead to potential tissue damage, thereby implying that extended exposure times could diminish therapeutic efficacy. [136] Secondly, considering the varying depths of ultrasound propagation in soft and hard tissues, the compositional differences between target and adjacent tissues may influence biological responses. [131]

## 4 Multi-stimuli-responsive materials

Current research predominantly focuses on single-stimulus-responsive systems, while the complex oral environment demands multi-signal coordination. Endogenous stimulus-responsive systems leverage unique physiological microenvironmental changes in lesioned areas to achieve spatially specific release of therapeutic agents, thereby minimizing adverse effects on healthy tissues. However, the intensity and duration of endogenous stimuli may be compromised by dynamic oral conditions such as salivary flow and dietary activities, which limit the temporal precision of these systems. [26,137] In contrast, exogenous stimulus-responsive nanomaterials enable drug release triggered by external physical stimuli at preprogrammed time intervals, offering more precise control mechanisms. Nevertheless, the penetration depth and spatial resolution of exogenous stimuli are also limited, which could compromise the targeting accuracy of drug delivery to lesioned areas. [78,89,131] To address the constraints of single-stimulus systems, researchers are developing integrated platforms that synergistically combine multiple stimulus-responsive mechanisms for periodontitis therapy.

**Fig. 12:**
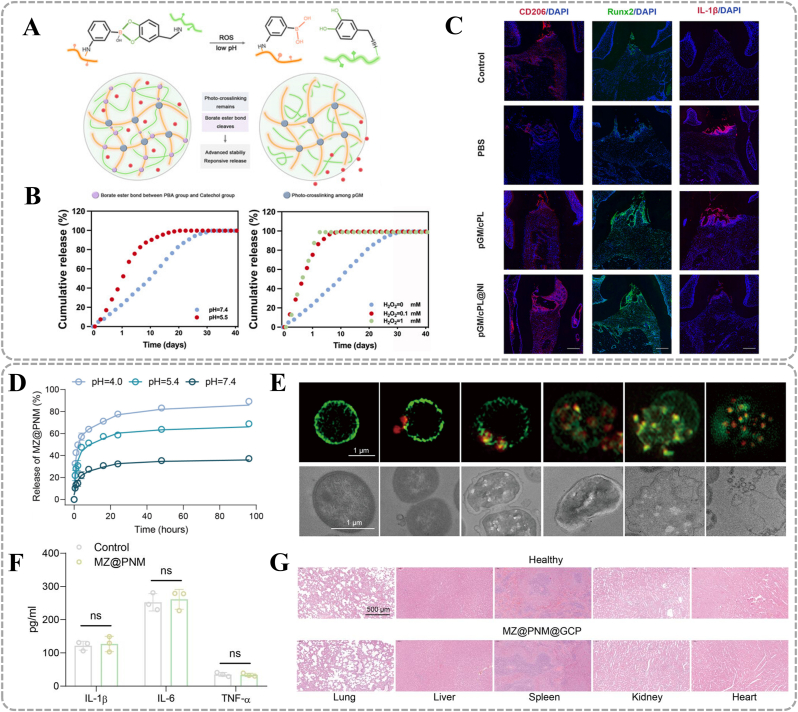
Multi-stimuli-responsive materials. (A) The stimulus-responsive mechanism of pGM/cPL@NI. (B) Cumulative release of MCC950 from pGM/cPL@NI under different pH and H_2_O_2_ environments. (C) IF staining of CD206 (red), Runx2 (green), IL-1β (red), and DAPI (blue) in periodontal tissue. (A, B, C) Reproduced with permission. [138] Copyright 2024, The Author(s). (D) MZ@PNM release curves of MZ@PNM@GCP under different pH conditions with time. (E) Representative fluorescence and TEM images of the destruction of P.g. by MZ@PNM. (F) Serum levels of IL1-β, IL-6 and TNF-α. (G) H&E staining of major organs in rats after MZ@PNM@GCP treatment. (D, E, F, G) Reproduced with permission. [140] Copyright 2022, American Chemical Society. (For interpretation of the references to colour in this figure legend, the reader is referred to the Web version of this article.)

For example, Liu et al. developed a multifunctional dual-crosslinked hydrogel (pGM/cPL@NI) to promote inflammatory resolution and bone regeneration by inhibiting NLRP3 in periodontitis. [138] The PBA-modified GelMA (pGM) and catechol-functionalized ε-PL (cPL) are crosslinked via dynamic boronate ester bonds. Under the low pH of periodontal lesions, hydrolysis of boronate ester bonds triggers the release of MCC950. Additionally, accumulated ROS at the lesion site oxidize catechol groups, disrupting the dynamic network and accelerating drug release (Fig. 12A). Although single endogenous stimuli may be unstable, synergistic action of multiple endogenous signals enhances material robustness and reliability. At neutral pH (7.4), the hydrogel exhibits slow drug release. In acidic (pH 5.5) or ROS-rich environments, release rates significantly increase (complete within 2 days), confirming stimulus responsiveness (Fig. 12B). MCC950, a specific NLRP3 inflammasome inhibitor, blocks downstream pro-inflammatory signaling pathways. In rat periodontitis models, the pGM/cPL@NI group showed upregulated expression of M2 macrophage marker (CD206) and osteogenic marker (Runx2), alongside reduced IL-1β levels (Fig. 12C). Additionally, a pH/light dual-responsive graphdiyne-iron nanozyme (GDY-Fe@HA-DA) with antibacterial and anti-inflammatory functions has been developed for periodontitis treatment. [139] Under 808 nm NIR laser irradiation, GDY rapidly elevates local temperature to 45–50 °C via efficient photothermal conversion, exhibiting bactericidal effects against *E*. *coli*, *S*. *aureus*, *P. gingivalis*, and other pathogens. However, conventional PTT often requires high-power laser irradiation to achieve sufficient antibacterial efficacy, which risks thermal damage to adjacent healthy tissues. Notably, the iron component in GDY-Fe@HA-DA catalyzes hydrogen peroxide decomposition through Fenton reactions, continuously generating highly reactive hydroxyl radicals (·OH). This chemodynamic therapy (CDT) synergizes with PTT to reduce the laser energy required for monotherapy, effectively mitigating thermal injury risks. Furthermore, pure photo-responsive materials are limited by insufficient penetration depth. CDT operates without requiring external light exposure and enables autonomous therapeutic action in deep tissues inaccessible to light, thereby compensating for the limitations of PTT. In vitro antibacterial assays demonstrated significantly enhanced bactericidal efficiency of the combined therapy compared to single-modality treatments, particularly against anaerobic pathogens like *P. gingivalis*. Moreover, a multi-responsive nanotherapeutic agent (MZ@PNM@GCP) has shown potent efficacy in modulating local immunity and eradicating pathogenic bacteria. [140] β-GP of GCP in the pre-gel state screens the cationic charges of chitosan's amino groups, weakening intermolecular electrostatic repulsion to maintain a sol state. Upon heating to 37 °C, β-GP's charge-shielding effect diminishes, allowing chitosan chains to crosslink via hydrophobic interactions and hydrogen bonds, forming a gel. After injection into periodontal pockets, MZ@PNM@GCP undergoes immediate sol-gel transition, preventing salivary washout or off-target diffusion while establishing a stable drug reservoir. In vivo release kinetics demonstrate sustained drug release from MZ@PNM@GCP over 16 days, significantly reducing dosing frequency. Under the acidic microenvironment of periodontitis, protonation of chitosan's amino groups disrupts the hydrogel's hydrogen-bonded network, triggering burst release of MZ. Localized, targeted drug delivery decreses systemic antibiotic overuse, thereby delaying the development of bacterial resistance.The GCP hydrogel releases 83 % of encapsulated MZ@PNM at pH 4.0, with markedly reduced release under neutral conditions (Fig. 12D). The released MZ@PNM directly targets the cell membrane of *P*. *gingivalis*, disrupting its structure. (Fig. 12E). The temperature/pH dual-responsive crosslinking system exhibits spatiotemporally precise therapeutic synergy. Thermoresponsive behavior ensures precise localization of therapeutics within periodontal pockets. pH-controlled release restricts drug activation to inflammatory microenvironments, effectively preventing off-target toxicity. In vivo safety assessments reveal normal serum biochemical parameters and unremarkable histopathological findings in major organs of the MZ@PNM treatment group (Fig. 12F) (Fig. 12G).

Multi-stimuli-responsive systems demonstrate significant potential for precision and multidimensional synergy in periodontitis therapy, although systematic studies on such platforms remain relatively scarce. Future research must delve deeper into their translational potential and undergo rigorous preclinical validation. However, multi-stimuli-responsive systems still face critical challenges that require urgent resolution. For instance, integrating multiple stimuli-responsive mechanisms relies on intricate material engineering, escalating synthetic complexity and costs. Additionally, multi-component systems heighten risks of immune activation or long-term biosafety concerns, particularly requiring thorough evaluation of degradation byproduct safety.

## 5 Harnessing responsive biomaterials for periodontitis

**Fig. 13:**
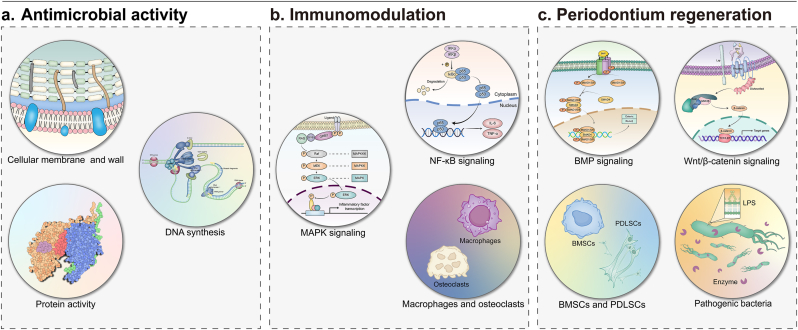
The active substances delivered by smart responsive materials modulate periodontal disease through three primary mechanisms: 1) Eradication of pathogenic microorganisms by disrupting bacterial cell membrane or wall structures, interfering DNA synthesis, and inhibiting protein function; 2) Immune modulation by regulating inflammatory signaling pathways (such as NF-κB and MAPK pathways) and controlling the activity of inflammatory cells (such as macrophages and osteoclasts); 3) Promoting regeneration of periodontal tissues by eliminating pathogenic bacteria and activating specific growth signaling pathways (such as BMP and Wnt/β-catenin pathways) and enhancing the proliferation and differentiation of BMSCs and PDLSCs. Abbreviation: NF-κB, Nuclear Factor kappa-light-chain-enhancer of activated B cells; MAPK, Mitogen-Activated Protein Kinase; BMP, bone morphogenetic protein; Wnt/β-catenin, Wingless-Int/β-catenin; BMSCs, bone marrow stem cells; PDLSCs, periodontal ligament stem cells.

In the treatment of periodontal disease, the application of smart responsive biomaterials provides an emerging platform for achieving precise disease control. The precise delivery of active substances based on intelligent responsive systems can block specific targets, thereby optimizing local therapeutic outcomes (Table .1). We will explore the potential applications of various stimuli-responsive biomaterials in the treatment of periodontal disease, including antibacterial, immunomodulatory, and periodontal tissue regeneration aspects (Fig. 13). By analyzing these strategies, we aim to elucidate effective pathways for the treatment of periodontal disease.

**Table 1:** Active substances used in the treatment of periodontal disease.

Type	Active substances	Mechanism	Ref
Inorganic agents	Ag	Antibacterial activity, inhibit peptidoglycan synthase activity	[128]
	Au-Ag	Immune modulation, block NF-κB signaling pathway	[80]
	Zn	Antibacterial activity, inhibit DNA polymerase activity	[141]
		Immune modulation, block NF-κB signling pathway	[142]
	ZnO	Antibacterial activity, destroy cell membrane	[117]
	Cu^+^	Antibacterial activity, inhibit protein synthesis	[143]
	Cu^2+^	Periodontium regeneration, promote BMSCs proliferation and differentiation	[143]
	CuS	Immune modulation, promote M2 macrophage polarization	[144]
		Periodontium regeneration, promote PDLSCs differentiation and collagen matrix synthesis	[113,114,145]
	CuTA	Immune modulation, blcok NF-κB signaling pathway	[39]
	Fe_2_O_3_	Antibacterial activity, destroy cell membrane	[102]
	CeO_2_	Immune modulation, block MAPK signaling pathway	[146]
	TiO_2_	Antibacterial activity, destroy cell membrane	[128]
	SrCl_2_	Periodontium regeneration, promote DPSCs differentiation	[147]
	Bi_2_Te_3_	Antibacterial activity, destroy cell membrane	[148]
	PB	Antibacterial activity, destroy cell membrane	[79,149]
	IR780 iodide	Antibacterial activity, destroy cell membrane	[104]
	NO	Antibacterial activity, oxidize DNA bases and amino acid residues	[150,151]
		Periodontium regeneration, promote collagen matrix synthesis	[150,151]
	H_2_O_2_	Antibacterial activity, destroy cell membrane	[152]
	H_2_S	Immune modulation, promote M2 macrophage polarization	[67]
		Periodontium regeneration, promote MSCs differentiation	[67]
Organic agents	MET	Immune modulation, clear ROS	[153]
		Periodontium regeneration, promote collagen synthesis, activate Wnt/β-catenin signaling pathway	[60,153]
	CIP	Antibacterial activity, inhibit DNA topoisomerases II and IV activity	[154]
	MZ	Antibacterial activity, destroy DNA bases	[140]
		Antibacterial activity, destroy cell membrane	[54,155,156]
	MINO	Antibacterial activity, inhibit protein synthesis	[29,102,157]
		Periodontium regeneration, inhibit osteoclast activity	[157]
	CEL	Immune modulation, inhibit COX-2 activity	[58]
	DOX	Antibacterial activity, inhibit protein synthesis	[158]
		Immune modulation, inhibit MMPs activity	[60]
	MH	Antibacterial activity, inhibit protein synthesis	[159]
	CHX	Antibacterial activity, destroy cell membrane	[95,151]
	TCS	Antibacterial activity, destroy cell membrane	[160]
	FLU	Immune modulation, inhibit cyclooxygenase activity	[160]
	MFX	Antibacterial activity, inhibit DNA topoisomerase II and IV activity	[94]
	ORN	Antibacterial activity, destroy cell membrane	[161,162]
	NAC	Immune modulation, block MAPK/NF-κB signaling pathway	[163]
		Periodontium regeneration, promote PDLSCs differentiation	[163]
	MCC950	Immune modulation, promote M2 macrophage polarization	[138]
	GSK-3β	Immune modulation, inhibit PI3K signaling pathway	[164]
		Periodontium regeneration, promote Wnt/β-catenin signaling pathway	[164]
	ALA	Periodontium regeneration, promote BMSCs differentiation	[165]
	PTB	Immune modulation, inhibit NF-κB signaling pathway	[166]
	SDF-1	Periodontium regeneration, promote PDLSCs and BMSCs differentiation	[38,153]
	BMP-2	Periodontium regeneration, promote MSCs differentiation	[58]
	BMP-7	Periodontium regeneration, promote MSCs differentiation	[162]
	EPO	Periodontium regeneration, promote HSCs proliferation	[167]
	bFGF	Periodontium regeneration, promote collagen matrix synthesis	[168]
	rhBMP-2	Periodontium regeneration, promote BMSCs differentiation	[169]
	Exosome	Immune modulation, promote M2 macrophage polarization	[92]
	ZIF-8	Antibacterial activity, induce DNA allostery	[142]
		Periodontium regeneration, promote rBMSCs differentiation	[142]
	Tbeta4	Immune modulation, promote M2 macrophage polarization	[56]
	TBO	Antibacterial activity, destroy cell membrane	[170,171]
	RB	Antibacterial activity, destroy cell membrane	[172]
	HMME	Antibacterial activity, destroy cell membrane	[173]
	Ce6	Antibacterial activity, destroy cell membrane	[127]
	QA	Antibacterial activity, destroy cell membrane	[158]
Natural extract	Eugenol	Antibacterial activity, destroy cell membrane	[174]
	EGCG	Antibacterial activity, destroy cell membrane, inhibit protein synthesis	[31]
		Immune modulation, inhibit MAPK/Mitf signaling pathway	
	BAI	Immune modulation, block NF-κB signaling pathway	[57,149,175]
		Periodontium regeneration, inhibit osteoclast activity	[174]
	CAPE	Immune modulation, inhibit macrophage M1 polarization	[176]
	QUE	Immune modulation, promote macrophage M2 polarization	[177]
	CS	Antibacterial activity, destroy cell membrane	[79,160]
		Periodontium regeneration, promote PDLSCs proliferation and adhesion	[145]
	TMC	Antibacterial activity, destroy cell membrane	[158]
	Gen	Immune modulation, inhibit NF-κB signaling pathway	[178]
	GA	Periodontium regeneration, promote Wnt/β-catenin signaling pathway	[21]
	TA	Antibacterial activity, destroy cell membrane	[179]
		Immune modulation, inhibit IL-6 and TNF-α expression, promote M2 macrophage polarization	[179,180]
	DHA	Antibacterial activity, destroy cell membrane	[170]
	Cur	Immune modulation, inhibit IL-6 and TNF-α expression	[181]
	NAR	Antibacterial activity, destroy cell membrane	[182]

Abbreviation: MET, metformin; CIP, ciprofloxacin; MZ, metronidazole; MINO, minocycline; MH, minocycline hydrochloride; CEL, celecoxib; DOX, doxycycline; CHX, chlorhexidine; TCS, triclosan; FLU, flurbiprofen; MFX, moxifloxacin; ORN, ornidazole; NAC, N-acetylcysteine; PTB, N-phenacylthiazolium bromide; BMP, bone morphogenetic protein; EPO, erythropoietin; bFGF, basic fibroblast growth factor; ZIF-8, zeolitic imidazole framework-8; HMME, hematoporphyrin monomethyl ether; QA, quaternary ammonium; CS, chitosan; TMC, N, N, N-trimethyl chitosan; TA, tannic acid; DHA, dihydroartemisinin; GA, gallic acid; Gen, genistein; Cur, curcumin; NAR, naringin; CAPE, caffeic acid phenethyl ester; EGCG, epigallocatechin gallate; QUE, quercetin; BAI, baicalein; COX-2, cyclooxygenase-2; MMPs, matrix metalloproteinases; PDLSCs, periodontal ligament stem cells; IL, interleukin; TNF, tumor necrosis factor; BMSCs, bone marrow stem cells; MSCs, mesenchymal stem cells; HSCs, hematopoietic stem cells; DPSCs, dental pulp stem cells; rBMSCs, rat bone mesenchymal stem cells; ROS, reactive oxygen species; NF-κB, Nuclear Factor kappa-light-chain-enhancer of activated B cells; MAPK, Mitogen-Activated Protein Kinase; Mitf, Microphthalmia-Associated Transcription Factor; Wnt/β-catenin, Wingless-Int/β-catenin; PB, prussian blue; Ce6, chlorin e6; TBO, toluidine blue O; RB, rose bengal; Tbeta4, thymosin β4; ALA, α-lipoic acid.

### 5.1 Smart responsive materials for antimicrobial therapy

#### 5.1.1 Disrupting cellular membrane and wall structures

In the progression of periodontal disease, the cell walls and membranes of periodontal pathogens are essential for their survival. These structures serve as the first line of defense, effectively resisting host immune responses and the invasion of medications. [183] Additionally, bacteria bridge to each other through surface molecules, promoting adhesion between bacteria and biofilm formation, effectively resisting salivary rinsing and mechanical removal. [184] Consequently, utilizing smart responsive materials to target and disrupt these structures has emerged as a promising strategy in periodontal therapy.

Hu et al. employed pH-responsive NPs to deliver quaternary ammonium (QA) aimed at inhibiting the formation of periodontal biofilms. [158] The low pH environment of periodontal inflammation triggers the degradation of these materials and the specific release of the drug. Released QA bind to anionic groups on the bacterial cell membrane, compromising its integrity and causing cellular contents to leak out, ultimately leading to bacterial death. Furthermore, Xin and colleagues designed an ultrasound-responsive multifunctional nanoplatform co-loaded with sonosensitizers and Ag to combat periodontal inflammation. [128] Under ultrasonic mediation, silver ions are precisely delivered to inflamed tissues, inhibiting the activity of enzymes involved in PGN synthesis and disrupting the cell wall structure. [185] Additionally, the incorporation of silver ions enhances the efficiency of ROS generation by sonosensitizers, synergizing antibacterial chemodynamic and sonodynamic effects. [186] Moreover, Ding et al. developed a thermosensitive hydrogel for the delivery of eugenol to treat periodontitis in rats. [174] Eugenol is capable of penetrating the cell membrane of *P. gingivalis*, irreversibly damaging the integrity of the cell membrane. [187] The thermosensitive hydrogel ensures sustained adhesion to periodontal sites and the controlled release of antimicrobial agents to enhance drug infiltration rates. Furthermore, a photocatalytic material, Bi_2_S_3_/Cu-TCPP, generates antibacterial ROS through light-induced electron-hole pairs reacting with H_2_O and O_2_. [87] ROS are capable of oxidizing lipid bilayers, thereby compromising the structural integrity of cell membranes. SEM revealed severe shrinkage and deformation of bacterial cell membranes (*e.g. P*. *gingivalis*) post-treatment. Additionally, a composite piezoelectric material, ZnO@Bdello, targets periodontal pathogens via dual mechanisms. [117] Bdellovibrio selectively lyses Gram-negative bacteria such as *P*. *gingivalis*, by targeting their cell walls. Similarly, mechanical stress from high-speed collisions between ZnO@Bdello and bacteria activates piezoelectric effects and catalytic ROS generation. Notably, piezo-photocatalytic technology integrates piezoelectric and photocatalytic principles, utilizing piezoelectric fields to enhance the separation efficiency of photogenerated charge carriers, thereby significantly boosting ROS generation capacity. This technology has been systematically reviewed in the field of antimicrobial applications. However, its specific application in periodontitis therapy remains unexplored. [111]

#### 5.1.2 Interfering DNA synthesis

DNA synthesis is fundamental to bacterial proliferation and is critical to the onset and progression of periodontal disease. Pathogens achieve rapid multiplication and biofilm formation through efficient DNA synthesis, thereby enhancing their colonization within the periodontal niche. [188] Given this, interfering with bacterial DNA synthesis to inhibit their proliferation has become an important strategy in treating periodontal disease. Utilizing intelligent responsive materials for the delivery of functional reagents offers additional strategies to achieve this goal.

Beg et al. designed thermosensitive NPs for the delivery of MFX to combat bacterial periodontal disease. [154] MFX inhibits the normal replication of bacterial DNA by targeting DNA topoisomerases II and IV. [189] This thermosensitive delivery system ensures prolonged retention of the drug within periodontal pockets, thereby enhancing therapeutic efficacy. Additionally, Mou et al. developed a pH-responsive nanohydrogel loaded with Zn^2+^ to inhibit the activity of *P. gingivalis*. [141] At low pH, Zn^2+^ is released, competing with metal ions required by DNA polymerases (such as Mg^2+^), thereby affecting normal DNA expression. [190] Furthermore, the introduction of Zn^2+^ enhances the stability of the hydrogel material, aiding in resistance to enzymatic degradation. Similarly, Yan et al. employed a pH-responsive hydrogel encapsulating MZ to block bacteria-mediated immune evasion. [140] MZ is activated by reductive enzymes within anaerobic bacteria, generating radicals that disrupt DNA bases. [191] Additionally, biomimetic Toll-like receptors (TLRs) on the surface of the material can block complement component 5a (C5a) secreted by *P. gingivalis*, thereby preventing its disruption of the periodontal immune response. Beyond disrupting cell membrane integrity, photocatalytic technology also targets bacterial DNA. Zhou et al. developed a photocatalyst, TBSMSPy^+^, capable of damaging the DNA structure of *P*. *gingivalis* and interfering with its replication processes. [88] Upon photoactivation, TBSMSPy^+^ generates stable free radicals that directly bind to bacterial DNA. The comet assay showed that the stained tails of bacteria incubated with irradiated TBSMSPy were all clearly presented, indicating efficient DNA damage. Concurrently, TBSMSPy^+^ catalyzes NADH oxidation, thereby blocking ATP synthesis and cutting off the energy supply required for DNA replication.

#### 5.1.3 Inhibiting protein activity

During the progress of periodontal disease, various proteins secreted by periodontal pathogens play a crucial role. These proteins, including collagen-binding proteins and membrane proteins, facilitate bacterial adherence to periodontal tissues and the formation of biofilms. [192] Additionally, bacterial protein products such as pili, hemolysin, and gingival protease act as toxin molecules that trigger periodontal inflammatory responses and mediate tissue damage. [193] Consequently, developing intelligent responsive materials which are capable of targeting bacterial proteins is especially necessary.

Tong et al. utilized ferromagnetic NPs to deliver MINO, successfully eliminating periodontal pathogenic biofilms in a rat model. [102] Guided by a magnetic field, the NPs can penetrate biofilm and reach the target area, releasing the antibiotic precisely. Released MINO binds to the 30S subunit of bacterial ribosomes, interfering with the pairing process between mRNA and tRNA, thereby preventing protein synthesis. [103] Moreover, apart from MINO, other tetracycline antibiotics such as DOX and MH also regulate the protein synthesis of periodontal pathogens. They share the same mechanism and are used in different responsive materials to exert their antibacterial effects. [158,159] For instance, Li et al. developed an enzyme-responsive membrane for controlled release of MH and localized periodontal therapy. [159] The polyphosphoester in the enzyme-responsive membrane is cleaved by ALPs to release MH, which disrupts bacterial protein synthesis. Additionally, the chitosan component of enzyme-responsive membrane interacts with bacterial membranes via electrostatic interactions, compromising membrane integrity, although the intrinsic antibacterial activity of chitosan alone is limited. [194] In addition to the aforementioned strategies, researchers are also exploring other methods to regulate bacterial protein activity. For example, Xu et al. designed a photosensitive membrane for the treatment of periodontal bacterial infections. [143] Under NIR light irradiation, the photothermal effect generated by Cu_2_O leads to widespread protein denaturation within cells, rendering them dysfunctional. Furthermore, a ROS-responsive system has been developed to precisely deliver EGCG for the antimicrobial treatment of diabetic periodontitis. [31] Research has demonstrated that EGCG can bind to collagenase secreted by bacteria and alter its conformation, thereby inhibiting its destructive effects on periodontal tissues. [195] Furthermore, a biodegradable piezoelectric membrane with enhanced antibacterial properties has been developed for periodontitis therapy. [196] The piezoelectric material PLLA-ZnO generates surface charges under ultrasonic stimulation, which disrupts bacterial membrane integrity and thereby inhibits bacterial activity. ZnO continuously released Zn^2+^ during degradation, which exerted antibacterial effects by inhibiting the activities of DNA replicases or respiratory chain-related enzymes.

### 5.2 Smart responsive materials for immunomodulation therapy

#### 5.2.1 Signal pathway modulation

**NF-κB signaling pathway** The NF-κB signaling pathway represents a critical regulatory mechanism for inflammation and occupies a central position in the onset and progression of periodontal inflammation. Typically, NF-κB remains inactive within cells due to its interaction with the inhibitory protein IκB. [197,198] Upon exposure of periodontal tissues to bacterial components, such as lipopolysaccharide (LPS), or other stimuli, IκB becomes phosphorylated and subsequently degraded, releasing NF-κB to translocate into the nucleus. [197] Translocated NF-κB binds to specific gene loci, activating the transcription and expression of pro-inflammatory genes, including IL-1β, IL-6 and TNF-α. [198] These pro-inflammatory mediators directly contribute to the degradation of the periodontal ECM; they also recruit inflammatory cells like macrophages and neutrophils, exacerbating the local inflammatory response. [199,200]

Given the pivotal role of the NF-κB signaling pathway in periodontal inflammation, numerous studies have proposed the use of intelligent responsive materials in drug delivery systems to precisely modulate its activity. Liu et al. developed a photo-sensitive composite hydrogel loaded with metal-organic frameworks that exhibited injectability and photopolymerization under ultraviolet light irradiation. [142] By continuously releasing Zn^2+^, the composite hydrogel reduces the activation of NF-κB signaling pathway and downregulates the expression of inflammation-related target genes, thereby alleviating periodontal inflammatory bone defects in rats. [201] Additionally, Li et al. encapsulated the NF-κB signaling pathway inhibitor, BAI, within CPD-capped MSNs for precise modulation of periodontal immune-inflammatory responses. [57] CPD layer controlled the GSH-responsive release of BAI; furthermore, the CPD coating facilitated cellular internalization of the nanosystem via endocytosis and thiol-mediated mechanisms, enhancing drug infiltration. Notably, increasing attention has been given to the interaction between NF-κB signaling and other critical pathways, including Phosphatidylinositol 3-Kinase/AKT (PI3K/AKT), Nuclear Factor-Erythroid 2-Related Factor 2 (Nrf2), Mitogen-Activated Protein Kinase (MAPK), and Wingless-Int (Wnt) signaling pathway, with the aim of identifying potential regulatory targets to enhance periodontal immune remodeling. For instance, Wang et al. utilized MPNs cross-linked with branched Au-Ag NPs to induce photothermal antibacterial and immunomodulatory effects for the synergistic treatment of periodontal disease. [80] The MPN enhances the photothermal properties of the Au-Ag NPs, achieving effective photothermal antibacterial activity against periodontal pathogens. Additionally, the procyanidins loaded onto the Au-Ag NPs can activate the PI3K/AKT signaling pathway to upregulate Nrf2, thereby inhibiting the NF-κB signaling pathway. [202] Similarly, Xu et al. developed a microenvironment-responsive metal-phenolic nanozyme release platform to control periodontal inflammation. [39] Negatively charged composite materials could remain at positively charged inflammatory sites via electrostatic adsorption and be hydrolyzed in response to increased MMP levels in periodontitis, achieving on-demand release of CuTA nanozymes. CuTA nanozymes could regulate the anti-inflammatory phenotypic polarization of macrophages and inhibit the secretion of pro-inflammatory factors via the Nrf2/NF-κB pathway, thereby alleviating inflammation and accelerating tissue regeneration in periodontitis.

**MAPK signal pathway** The MAPK signaling pathway is another critical pathway that plays a key role in the initiation and progression of periodontal inflammation. Within the periodontal inflammatory environment, bacteria and their toxins, such as PGN, LPS, and lipoproteins, interact with pattern recognition receptors on the surface of host cells, such as TLRs, triggering the transduction of the MAPK signaling pathway. [203] This process involves sequential phosphorylation and nuclear translocation of Extracellular signal-Regulated Kinases (ERK), JNK, and p38 MAPK. [204] The activated kinases promote the activation of related transcription factors, leading to the production of pro-inflammatory cytokines such as IL-4, IL-6, and IL-8, which attract macrophages and osteoclasts, exacerbating local inflammation. [[205], [206], [207], [208]] In addition, upregulation of the MAPK pathway also promotes the expression of genes encoding MMPs (such as MMP-9 and MMP-13), accelerating alveolar bone resorption and damaging periodontal support tissues. [209,210]

Recent studies have targeted the MAPK signaling pathway for the treatment of periodontal inflammation, utilizing intelligent responsive materials to achieve precise targeted therapy. Yu et al. introduced a therapeutic approach using in situ injection of CeO_2_ NPs for the treatment of periodontitis. [146] CeO_2_ NPs act as antioxidant enzymes, responding to and scavenging excessive ROS accumulated at inflamed sites, thereby inhibiting the activation of the MAPK signaling pathway. Notably, the inhibition of MAPK also affects the downstream transduction of the NF-κB signaling pathway, further suppressing the expression of local inflammatory factors. Moreover, Qiu et al. developed a ROS-responsive nanoplatform Pssl-NAC to remodel periodontal immune microenvironment. [163] Similarly, Pssl-NAC-mediated ROS clearance can effectively downregulate the MAPK/NF-κB signaling pathway and increase the expression of anti-inflammatory signals such as IL-4 and IL-13, thereby promoting macrophage polarization towards the M2 anti-inflammatory phenotype. Furthermore, Chanaj-Kaczmarek and colleagues designed a thermosensitive hydrogel to modulate aberrant enzymatic activities in periodontal disease. [175] The thermosensitive phase-transition carrier emulsion effectively penetrates narrow periodontal pockets, enhancing the infiltration of BAI and CS. BAI inhibits the MAPK signaling pathway, reducing the secretion of pro-matrix metalloproteinase-1 (pro-MMP-1) and MMP-3. [211] Meanwhile, CS inhibits hyaluronic acid activity, maintaining the structural integrity of the periodontal ECM.

#### 5.2.2 Cell fate manipulation

**Macrophage** In periodontal disease, the phenotype of macrophages undergoes transformation influenced by various factors, which directly impacts the progression and final outcome of inflammation. Periodontal disease is a localized inflammatory reaction triggered by dental plaque biofilm, wherein bacteria and their components, such as LPS, peptidoglycan (PGN), and flagellin, can activate macrophages and promote their transition to the pro-inflammatory M1 subtype. [205] M1 macrophages release large quantities of pro-inflammatory cytokines (such as IL-6, IL-17, and IL-18) along with ROS and reactive nitrogen intermediates, thereby exacerbating the local inflammatory response. [212,213] However, during the terminal stages of inflammation or under the stimulation of specific signals, macrophages may shift towards the anti-inflammatory M2 phenotype. [214] M2 macrophages possess the capability to secrete anti-inflammatory cytokines (such as IL-4, IL-10, and TGF-β) and also facilitate the proliferation of fibroblasts and the deposition of collagen, all of which play a crucial role in reducing inflammation and promoting tissue regeneration. [212]

Currently, strategies employing intelligent responsive materials to inhibit M1 macrophage activity or induce polarization towards an M2 phenotype have been extensively explored for reshaping the periodontal immune microenvironment. Liu et al. developed an environmentally responsive hydrogel for the delivery of NLRP3 inhibitors aimed at promoting the resolution of inflammation. [138] In periodontitis, bacterial components activate the NLRP3 inflammasome, leading to the maturation and release of IL-1β and IL-18, which in turn activate M1 macrophages. [199] In environments rich in ROS or acidic conditions, the drug delivery system responsively releases NLRP3 inhibitors, thereby downregulating the activation of M1 macrophages and attenuating pro-inflammatory polarization and cytokine cascades. Similarly, Peng and colleagues utilized thermosensitive NPs for the local delivery of caffeic acid phenethyl ester (CAPE) to treat periodontitis. [176] With the rise in oral temperature, the NPs gradually release CAPE, inhibiting the activation of M1 macrophages and consequently reducing the local expression of inflammatory cytokines such as TNF-α, IL-1β, IL-6, and IL-17. [215] Notably, recent research trends not only focus on the inhibition of M1 macrophages but also explore the reprogramming of M1 to M2 macrophages. Yang et al. developed CuS NPs to promote immunoreprogramming in periodontal disease. [144] Under spatiotemporal manipulation by NIR light, CuS NPs can precisely release functional agents to induce M2 polarization of macrophages, mediating an anti-inflammatory response. Additionally, these materials can kill bacteria through NIR light-triggered PTT, synergistically alleviating periodontal damage. Likewise, Xu et al. utilized thermosensitive organic frameworks for the delivery of quercetin (QUE) to treat periodontitis in rats. [177] The study indicated that QUE upregulates factors such as IL-3 and IL-10, activating the STAT signaling pathway, thus promoting the polarization of macrophages into the M2 phenotype. [216] The thermosensitive organic framework provides excellent fluidity and stability post-injection, resisting washout by oral fluids and achieving efficient drug retention.

**Osteoclast** Accumulated macrophages and T lymphocytes within the inflammation microenvironment secrete a range of pro-inflammatory cytokines, including IL-1, TNF-α, Macrophage Colony-Stimulating Factor (M-CSF), and Receptor Activator of Nuclear Factor-kappa B Ligand (RANKL), which promote the activation and accumulation of osteoclasts. [217] Osteoclasts accelerate alveolar bone resorption and the destruction of supporting soft tissues around teeth by secreting acidic substances and proteases such as MMPs. Moreover, osteoclasts secrete additional pro-inflammatory factors, such as PGE2, IL-8, and IL-17, further exacerbating the inflammatory response and potentially promoting the differentiation of more osteoclasts, thereby establishing a vicious feedback loop. [218]

With advancing research, the use of various responsive materials to precisely regulate osteoclast activity is providing new strategies for the treatment of periodontal disease. Chen et al. designed a ROS-sensitive oral barrier membrane to alleviate periodontal inflammation and bone tissue destruction. [157] Enriched ROS in the local microenvironment triggers the cleavage of borate ester bonds within the material, leading to the release of MINO. MINO can reduce the production of inflammatory factors such as IL-1β, IL-6, and TNF-α, inhibiting osteoclast-mediated inflammatory damage. [219] Additionally, Ding et al. utilized a thermosensitive microemulsion-gel composite to deliver BAI for the modulation of periodontal inflammation. [174] The microemulsion-gel composite releases BAI over an extended period at higher temperatures, suppressing RANKL mRNA expression and downregulating osteoclast differentiation. Further investigation revealed that the downregulation of RANKL mRNA may be associated with the crosstalk between multiple signaling pathways mediated by BAI. BAI can inhibit the expression of TLR2 and TLR4 and their downstream signaling pathways, such as NF-κB, p38 MAPK, and JNK, thereby reducing the expression of osteoclast activation factors. [220] Similarly, Feng et al. constructed a ROS/glucose-responsive EGCG release system, effectively treating periodontal inflammation in diabetic rats. [31] EGCG can suppress the expression of inflammatory cytokines such as IL-17 and IL-1β by inhibiting the MAPK/Microphthalmia-Associated Transcription Factor (Mitf) signaling pathway, thereby reducing inflammatory bone resorption. [221] Notably, EGCG forms phenylboronate ester bonds with PBA in the material, conferring ROS/glucose sensitivity to the system.

### 5.3 Smart responsive materials for periodontium regeneration therapy

#### 5.3.1 Signal pathway modulation

**BMP signaling pathway** The BMP signaling pathway is essential for tissue regeneration. This pathway activates intracellular Smad proteins through the binding of BMP ligands to their cell surface receptors, subsequently regulating the expression of downstream genes. The transcription of downstream genes such as Runx2, Osterix, and Col1A1 can induce the differentiation of fibroblasts and osteoblasts, thereby promoting the synthesis and mineralization of the ECM. [222] However, inflammatory mediators such as IL-6 and IL-10 can inhibit BMP signaling by promoting the phosphorylation and degradation of Smad proteins. [223,224] Additionally, oxidative stress products generated in the inflammatory microenvironment, such as ROS and RNS, can oxidize and disrupt the amino acid residues of key signaling molecules, thereby weakening the function of the BMP signaling pathway. [225] The inhibitory effects of pro-inflammatory factors and oxidative stress products on BMP signaling hinder its positive regulatory effects in the regeneration of periodontal tissues.

Considering the importance of the BMP signaling pathway in periodontal tissue regeneration, progress has been made in exploring new approaches using various stimuli-responsive materials for targeted modulation. Zhao et al. utilized a polyethyleneimine (PEI)-based hydrogel to achieve targeted delivery of Metformin (MET) in a chronic periodontal inflammation model of diabetic rats. [60] Research indicates that MET enhances BMP-2 expression, activates ALPs activity, and promotes the synthesis of collagen matrix, demonstrating its efficacy in reducing inflammation and periodontal bone loss. [226] Especifically, the B-N coordination bond between the drug and the PBA-PEI carrier responds to the level of ROS in the periodontal microenvironment, enabling targeted drug release and specific treatment of periodontal inflammation. Additionally, Hao et al. designed a dual-responsive core-shell structure combining thio-modified MSNs clusters with porous PLGA microparticles for the co-delivery of BMP and CEL, offering a new strategy for periodontal disease treatment. [58] After injection into the periodontal inflammatory area, MMPs in the inflammatory microenvironment trigger the disassembly of MSNs clusters, releasing BMP-2 to promote bone regeneration. The inner core of the MSNs enters cells via endocytosis and releases antibiotics in response to GSH to suppress inflammation. Concurrently, the porous PLGA microparticle scaffold provides a biomimetic structure similar to the ECM, synergistically promoting bone regeneration. Furthermore, Wang et al. developed thermosensitive microparticles for the delivery of genistein (Gen) to promote inflammation resolution and osteogenic differentiation in periodontitis. [178] Gen inhibits the activation of inflammatory signaling pathways such as NF-κB, reducing the expression of pro-inflammatory cytokines (such as TNF-α, IL-1β, and IL-6), thus helping to restore the activity of the BMP signaling pathway and osteogenic regeneration. [227] The microparticles serve as carriers for Gen delivery, thickening and adhering in response to thermal stimuli in the oral cavity, effectively resisting saliva washing and ensuring local effective drug administration.

**Wnt/β-catenin signaling pathway** In the regeneration of periodontal tissues, alongside the BMP signaling pathway, the Wnt/β-catenin signaling pathway also plays a critical role. This pathway functions through the binding of Wnt ligands to Frizzled receptors, inhibiting the degradation of β-catenin, leading to its accumulation in the nucleus where it binds to Transcription factor/Lymphoid Enhancer Binding Factor (TCF/LEF) transcription factors. [228] This process activates the expression of genes that regulate the proliferation and differentiation of fibroblasts and osteoblasts, contributing to the reconstruction of the periodontal ligament and alveolar bone. [229] However, in an inflammatory environment, inflammatory factors such as IL-1β and TNF-α can interfere with the Wnt/β-catenin signaling pathway by enhancing the activity of glycogen synthase kinase-3 beta (GSK-3β), promoting the degradation of β-catenin and inhibiting its nuclear accumulation. [230,231] Additionally, inflammatory factors may affect the expression of Wnt ligands or the functionality of their receptors, weakening signal transmission. [232] The inhibition of the Wnt/β-catenin signaling pathway in an inflammatory context leads to a decline in periodontal repair capabilities and accelerates the resorption of alveolar bone. [233]

Given the critical role of the Wnt/β-catenin pathway in periodontal tissue regeneration, numerous targeted therapeutic approaches have been proposed to restore the activity of this signaling pathway. Yosif Almoshari et al. designed a bone-directed thermosensitive hydrogel loaded with the GSK3 inhibitor 6-Bromoindirubin-3′-oxime (BIO), which effectively mitigates periodontal tissue damage associated with experimental periodontitis. [164] The thermosensitive hydrogel adheres to periodontal hard tissues and gradually release BIO to exert its osteogenic effects locally, effectively avoiding the potential side effects of prolonged pathway activation. Additionally, Luo et al. developed a dynamic hydrogel-metallic organic framework system for the controlled delivery of gallic acid (GA) to promote bone regeneration in periodontitis. [21] GA activates the canonical Wnt/β-catenin signaling pathway, upregulating the activity of ALPs and the expression of collagen, demonstrating significant osteogenic effects. [234] The dual-crosslinked network is sensitive to the pH and ROS levels in the inflammatory environment, overcoming the challenges posed by the dynamic and complex oral environment, including saliva flow, chemical components, and temperature fluctuations, to achieve precise drug delivery. Similarly, Wang and colleagues utilized thermosensitive elements-modified MSNs to deliver MET to restore the activity of the Wnt/β-catenin pathway. [153] Thermosensitive NPs slowly degrade to continuously release MET, clearing excess ROS produced under high-glucose conditions and reactivating the Wnt/β-catenin pathway, thereby reversing the inhibited osteogenesis in rat bone mesenchymal stem cells (rBMSCs). Interestingly, MET does not exert a definitive positive impact on the osteogenic differentiation of rBMSCs under normoglycemic conditions, suggesting an interaction between inflammatory microenvironments with high glucose and ROS levels and the Wnt/β-catenin pathway.

#### 5.3.2 Cell fate manipulation

**BMSCs**, encompassing MSCs and hematopoietic stem cells (HSCs), possess self-renewal capacity and multi-lineage differentiation potential. These cells can migrate to damaged sites and differentiate into various cell types, such as osteoblasts and chondrocytes, promoting the synthesis and mineralization of periodontal matrix. [235,236] Additionally, these stem cells secrete a variety of growth factors, including Vascular Endothelial Growth Factor (VEGF), TGF-β, Epidermal Growth Factor (EGF), and Insulin-like Growth Factor (IGF), which facilitate angiogenesis and regulate inflammatory responses. [237,238] However, in the context of periodontal disease, the accumulation of inflammatory mediators such as TNF-α, IL-1β, and PGE2 can interfere with the proliferation, differentiation, and migration of BMSCs, diminishing their ability to secrete beneficial growth factors and consequently affecting the regeneration of periodontal tissues. [[239], [240], [241]]

The utilization of intelligent responsive materials to specifically mobilize MSCs and HSCs provides new strategies for the repair of damaged tissues in periodontal disease. Zheng et al. developed a photosensitive film for guiding periodontal regeneration. [143] Based on a photocatalytic approach, the film material generates ROS upon exposure to blue light, promoting the oxidation of Cu ^+^ to Cu^2+^. Appropriate levels of Cu^2+^ enhance the expression of factors such as RUNX2 and osteocalcin (OCN), which are conducive to the proliferation and osteogenic differentiation of BMSCs. [242] Similarly, Wang et al. employed smart responsive NPs to deliver α-lipoic acid (ALA) for the treatment of periodontal inflammation. [165] Lipases in dental plaque degrade the material, releasing ALA, which effectively mitigates ROS-induced suppression of osteogenic differentiation in BMSCs. Beyond activating MSCs, several recent studies have focused on the role of HSCs in promoting periodontal regeneration. Roldán et al. utilized injectable piezoelectric fillers to enhance antibacterial efficacy and bone tissue regeneration in periodontal disease. [113] Piezoelectric BaTiO_3_ fillers generate electrical charges when subjected to biomechanical vibrations, killing periodontal pathogens and stimulating the activation of HSCs. Activated HSCs can promote angiogenesis, improving local microcirculation and providing essential nutritional support for periodontal tissue repair. [243] Similarly, Xu et al. designed a thermosensitive hydrogel for the delivery of erythropoietin (EPO) to achieve periodontal regeneration. [167] Especifically, the study highlighted that, in addition to mediating the proliferation of HSCs, EPO can inhibit osteoclast resorption function via the activation of the B2-EphB4 signaling pathway. [244]

**PDLSCs** exhibit a propensity to differentiate into osteoblasts and fibroblasts, playing a pivotal role in the regeneration of periodontal tissues. When PDLSCs differentiate into osteoblasts, they secrete BMP, TGF-β, osteopontin, and OCN, promoting the differentiation and maturation of new bone. [245,246] Upon differentiation into fibroblasts, they produce substantial amounts of collagen and other ECM components, such as fibronectin and laminin, aiding in the construction and maintenance of the periodontal ligament fibers. [247] However, mediators released during inflammation, such as TNF-α, IL-1β, and ROS, can inhibit the activation and differentiation of PDLSCs, weakening their positive role in periodontal tissue repair. [[248], [249], [250]]

The use of intelligent responsive materials to specifically mobilize PDLSCs and promote their osteogenic and fibrogenic differentiation offers new strategies for the regeneration of soft and hard tissues in periodontal disease. Qiu et al. utilized ROS-responsive NPs to remodel the periodontal inflammatory microenvironment to enhance osteogenesis. [55] High concentrations of ROS can induce mitochondrial dysfunction in PDLSCs, affecting their energy metabolism and osteogenic capacity. [251] The ROS-reactive cleavage of sulfoketal bonds in the NPs promotes polymer degradation and the release of ROS scavengers, thereby reversing the suppressed osteogenic differentiation of PDLSCs. Similarly, Liu et al. developed a gingipain-responsive hydrogel loaded with SDF-1 to promote periodontal tissue regeneration. [38] The hydrogel releases SDF-1 in response to gingipain stimulation, promoting the proliferation, migration, and osteogenic differentiation of PDLSCs. [252] As research advances, it has become evident that fibroblasts differentiated from PDLSCs also make significant contributions to periodontal regeneration. Houshyar et al. designed a CS/BaTiO_3_ composite membrane for the treatment of periodontal disease. [145] CS exhibits excellent affinity toward human PDLSCs, facilitating their migration and adhesion. [253] Meanwhile, the electrical impulses generated by the BaTiO_3_ piezoelectric scaffold stimulate the differentiation and proliferation of fibroblasts, mediating the reconstruction of the collagen matrix in damaged periodontal tissues. Similarly, Ji et al. used an injectable thermosensitive hydrogel to deliver basic fibroblast growth factor (bFGF) for the reconstruction of periodontal defects. [168] bFGF based on a thermosensitive system can be released in a sustained manner, continuously mediating the proliferation of fibroblasts and the deposition of periodontal collagen matrix.

#### 5.3.3 Promoting tissue regeneration through antibacterial effects

Periodontal pathogens destroy periodontal tissues through various mechanisms. They secrete proteases such as collagenase and elastase. These enzymes directly break down collagen fibers in gingival and periodontal ligaments. [254] Meanwhile, bacterial endotoxins like lipopolysaccharides (LPS) activate host immune cells. This triggers excessive secretion of inflammatory factors such as IL-1β and TNF-α. It accelerates the destruction of alveolar bone and periodontal ligaments. [255] Current research develops smart responsive materials combined with antibacterial and tissue regeneration strategies. They achieve dual effects: pathogen removal and repair promotion. This provides a new direction for periodontal disease treatment.

For instance, magnetic microspheres loaded with photosensitizers achieve precise antibacterial effects for bone defect healing. [104] *P. gingivalis* causes bone resorption by secreting proteases and endotoxins. [256] Functionalized microspheres (FMSs) are magnetically guided to infected areas. Under NIR light, they release ROS to kill bacteria. After pathogen elimination, bone resorption is inhibited, improving the microenvironment for new bone formation. Notably, BMP-2 alone promotes bone formation but shows lower efficacy than material groups due to ineffective bacterial suppression. This indicates that growth factors alone cannot counteract pathogens' negative impact on bone regeneration. Killing bacteria is crucial for promoting healing. Additionally, a ZIF-8-modified injectable photopolymerizing GelMA hydrogel offers synergistic antibacterial, anti-inflammatory, and tissue-regenerative functions. [142] GelMA-Z is liquid at body temperature, filling irregular periodontal pockets via injection. UV irradiation crosslinks lithium acylphosphinate (LAP) in GelMA, forming a stable porous gel network. This ensures material retention and sustained drug release. Persistent presence of periodontal pathogens activates host inflammation, such as the NF-κB pathway, inhibiting bone regeneration. After Zn^2+^ from ZIF-8 kills bacteria, the inflammatory microenvironment clears, facilitating alveolar bone regeneration. In a rat periodontitis model, GelMA-Z significantly reduced bacterial load in periodontal pockets. It lowered TNF-α and COX2 expression in gingival tissues, with bone resorption levels closest to healthy controls. These findings suggest that antibacterial action not only eliminates pathogens but also directly supports tissue regeneration by reducing inflammation and optimizing the local microenvironment.

## 6 Conclusions and prospects

This article reviews and discusses various strategies for constructing intelligent responsive materials for the treatment of periodontal disease. Endogenous physiological microenvironments (such as pH, blood glucose concentration, ROS levels, enzyme activities and bioelectrical activity) drive spatial-specific targeting of responsive materials, whereas exogenous stimuli (such as light, thermals, vibration, magnetic fields, and ultrasound) enable temporal control of treatment delivery. The materials designed can precisely deliver functional active substances in response to both endogenous and exogenous stimuli, specifically achieving antibacterial effects, immune modulation, and periodontal tissue regeneration, thus contributing to the treatment of periodontal disease (Table .2). Despite the significant potential and advantages demonstrated by intelligent responsive materials in the treatment of periodontal disease, future research must address several challenges.

**Table 2:** Smart stimuli-responsive materials for periodontal disease treatment.

Response stimulus		Material	Response strategy	Material characteristics	Ref
Endogenous	PH	TMC-Lip-DOX	Protonation reactions of amino groups	Favorable biocompatibility (79 % RGR for HPDLFs), effective antibacterial activity (>75 % inhibition against *P. gingivalis* and *P. intermedia*)	[158]
		CMCS	Imine bond formation with the 4-FPBA respond to PH cleavage	Good biocompatibility (non-toxic to RAW 264.7 and MC3T3 cells), effective antibacterial activity (>70 % inhibition against *P. gingivalis* and *A. actinomycetemcomitans*)	[21]
		CS	Protonation reactions of amino groups	High loading capacity (loaded with 96.33 % FLB and 93.67 % TCS nanoparticles), excellent bioadhesion (adhesion force: 137.22 ± 5.05 g)	[160]
		Au@MPN-BMP2	Protonation reactions of phenolic hydroxyl groups	High loading efficiency (BMP-2 loading efficiency >92 %), good biocompatibility (non-toxic to BMSCs, cell viability >90 %)	[20]
		PLGA/CS	Composite with pH-sensitive CS	High loading capacity (drug encapsulation efficiency: 54.5 ± 4.9 %), good biocompatibility (similar inflammatory levels and bone loss extent to untreated group)	[257]
		Mino-ZnO@Alb	Composite with pH-sensitive carboxyl groups	Good biocompatibility (gingival cell viability >85 %), excellent bioadhesion (maximum adhesion force: 0.35 N)	[141]
		DSPE-PEG/PAMAM	Amides bond responds to pH cleavage	High loading capacity (DLC: ALA 13.61 %; MINO 17.17 %), excellent biocompatibility (>90 % BMSCs viability)	[165]
		ZIF-8/PPEMA-GelMA	Protonation reactions of imidazole ligands groups	Excellent stability (compressive modulus ∼40 kPa, swelling ratio stabilized at ∼140 % after 2 h in PBS), good biocompatibility (HGFs and OBs viability >90 %)	[258]
	Glucose	CS/PEO	Allosteric effects based on glucose oxidase	High stability (intact in acetic acid for 24 h), superior mechanical properties (compressive strength:43.09 kPa; G':275.10 Pa)	[259]
		CaAlg@MINO/GOx/CAT/ZIF-8	Allosteric effects based on glucose oxidase	High chemical stability (GOx activity retained in Protease K-containing high-glucose solution), high loading capacity (GOx encapsulation efficiency: 90.12 %)	[29]
		PEO/c-PVA/HTCC	Allosteric effects based on glucose oxidase	High loading capacity (rhBMP-2 encapsulation efficiency: 90.16 % ± 3.3 %), low toxicity (H_2_O_2_ production is lower than the apoptotic threshold of BMSCs)	[169]
		Au/Pt NCs@GOx	Catalytic effects based on glucose oxidase	Excellent biocompatibility (hemolysis <5 %), low cytotoxicity (PDLCs viability >95 %)	[30]
		MSN-Au@CO	Composite with Au NPs	Good biocompatibility (did not produce obvious hemolysis), excellent biocompatibility (83.5 % hPDLCs viability)	[34]
	Enzyme	TM/BHT/CuTA	Ester linkage responsive to MMPs	Good biocompatibility (hemolysis ratios <5 %), high loading capacity (BHT:CuTA mass ratio 4:1)	[39]
		PLGA/MSNs-PMS	Composite with MMP-sensitive peptide	High loading capacity (drug loading: Celecoxib 7.4 mg/g; BMP-2 8.3 mg/g), high stability (drug leakage-free over 18 days under non-stimuli conditions)	[58]
		PPEM	Phosphate bond respond to ALPs cleavage	High flexibility (elongation at break: 55.30 % ± 0.04 %, tensile modulus: 83.1 ± 3.06 MPa), good bioadhesion (water contact angle: 51.9 ± 3.6°)	[159]
		PEG	Composite with MMP-sensitive peptide	Excellent biodegradability (complete degradation within 72 h), low cytotoxicity (HGFs viability >90 %)	[260]
		PEGPD@SDF-1	Anchor peptide respond to gingipain cleavage	Good Biocompatibility (non-toxic to PDLCs), excellent chemical stability (degradation time >20 days in PBS)	[38]
	Redox	PBA-PEI	B-N coordination bond formation with the drug respond to ROS cleavage	Superior adhesion (adhesive strength: 4.5 kPa), excellent biocompatibility (hemolysis <5 %)	[60]
		HQRB-SS-Dex	Dynamic borate ester bond formation with the 4-FPBA respond to ROS cleavage	Good biocompatibility (94 % HGFs viability), excellent stability (little difference on particles sizes from PH7.4 to PH5.5)	[172]
		MSN	Composite with GSH-sensitive disulfide bond	High loading capacity (BE loading: 11.5 %), good physicochemical stability (zeta potential: from 31.8mv to 21.6 mv within 4 days)	[57]
		CHX@PCL-PLG	Guanidinium group respond to ROS cleavage	Excellent biocompatibility (hemolysis ≤5 %), effective loading capacity (CHX loading: 33.8 ± 4.23 %)	[151]
		PLGA-TK-PEG	Thioketal bond respond to ROS cleavage	Excellent antibacterial activity (>85 % inhibition against *P.gingivalis*), superior mechanical strength (15 % PLGA bearing capacity: >0.3 N compressive force)	[54]
	Endogenous electricity	PGO-PHA-AG	Conductive effect	Superior mechanical strength (10 wt‰ PGO: 140 kPa compressive strength), excellent biodegradability (70 % degradation at PBS with collagenase II for 4 weeks), good electrical conductivity (10 wt‰ PGO conductivity:1.6 mS/cm)	[66]
		BNP-PEDOT-PSF	Conductive effect	Superior electrical conductivity (2 wt‰ PEDOT-PSF conductivity: 13.12 ± 1.66 mS/cm), excellent mechanical strength (compression strength:100 kPa)	[67]
		GMPA	Conductive effect	Excellent electrical conductivity (conductivity: 0.70 mS/cm), excellent mechanical strength (elastic modulus: 724.61 ± 35.03 Pa), good biodegradability (complete degradation within 28 days)	[69]
Exogenous	Light	AuAg@PC-Fe	Photothermal effect	Superior photothermal efficiency (rose to 50 °C within 3 min, 2.5 W/cm^2^ NIR), favorable biocompatibility (96 % fibroblasts viability)	[80]
		MPB-BA	Photothermal effect	Superior antibacterial properties (99.9 % inhibition against *F. nucleatum* and *P.gingivalis*), favorable biodegradability (95 % has been excreted at 72 h in feces)	[149]
		PEG-b-PArg	Composite with photothermocatalyst Bi_2_Te_3_	Excellent photothermal capacity (rose to 53.8 °C within 5 min, 1.0 W/cm^2^ NIR), Good biocompatibility (HGFs cell viability >90 %)	[148]
		Fe_3_O_4_-silane@Ce6/C6	Composite with photosensitizer C6	Good antibacterial performance (*P. gingivalis* CFU was reduced by more than 5 log), excellent biocompatibility (L929 fibroblast cells viability >90 %)	[261]
		TiO_2_@PDA	Photocatalytic effect	Excellent photothermal capacity (rose to 48.5 ± 0.71 °C within 30 min, 1.0 W/cm^2^ NIR), superior antibacterial properties (98.52 ± 0.78 % inhibition against *E. coli*)	[143]
		GelMA-Au NBPs@SiO_2_	Photothermal effect	Excellent biocompatibility (L929 fibroblast cells viability >80 %, within 5 min, 1.2 W/cm^2^ NIR), good photothermal ability (rose to 57.5 °C within 5 min, 1.2 W/cm^2^ NIR)	[262]
		PAA	Composite with photosensitizer TBO	Excellent release ability (68.26 % TBO released in 24 h), high stability (store for 6 weeks at PH 7.2–7.9)	[171]
	Magnetism	Fe_3_O_4_	Magnetization effect	Excellent biocompatibility (MC3T3-E1 cells viability >80 %), effective antibacterial properties (80 % inhibition against *C. albicans*)	[152]
		APTS@Fe_2_O_3_	Magnetization effect	Good antibacterial performance (89.62 ± 13.68 % inhibition against *P. gingivalis*), high loading capacity (IR780 EE: 66.57 ± 0.86 %, IR780 LE: 3.56 ± 0.09 %)	[104]
		Fe_3_O_4_@PEI/BiVO_4_	Composite with magnetic nanoparticle	Superior antibiofilm activity (destroy 88 % of biofilms on a abutment)	[105]
	Thermal	Curdlan	Temperature-induced conformational changes and hydrophobic interactions	Excellent biocompatibility (hPDLCs viability >80 %), effective antibacterial properties (74.4 % inhibition against *S. aureus*)	[95]
		PEO-PPO-PEO	Temperature-induced conformational changes and hydrophobic interactions	Superior biodegradability (at 30 % w/v concentration, completely eroded within 48 h), superior biocompatibility (at 25 % w/v, 84.5 % MC3T3-E1 cells viability)	[164]
		MSN-PDLLA-PEG-PDLLA	Temperature-induced conformational changes and hydrophobic interactions	Sustained release ability (80 % SDF-1 released in 2 weeks, 37 °C PBS), excellent biodegradability (gradually degrade within 4 weeks when implanted subcutaneously in SD rats)	[153]
		PLGA	Temperature-induced conformational changes and hydrophobic interactions	High loading efficiency (over 51.17 % drug loading), good mechanical properties (mean bioadhesion strength: 11.64 ± 0.07 g)	[154]
		CS	Composite with thermal-sensitive β-GP	Sustained release ability (94.2 % aspirin released in 8 days, 37 °C PBS), excellent biodegradability (degrade >80 % in 30 days, 37 °C PBS)	[167]
		CMCS	Temperature-induced conformational changes and hydrophobic interactions	Excellent biodegradability (degrade 80 % within 12 days in PBS), low toxicity (90 % RAW 264.7 and hPDLSCs viability)	[176]
	Vibration	ZnO@Bdello	Convert mechanical energy into electrical energy	High anti-biofilm capability (penetrate about 50 μm thick biofilm within 2 h), excellent biocompatibility (3T3 cells viability >80 % with 50 μg/mL ZnO attaching to10^7^ PFU/ml of *Bdellovibrio*)	[117]
		NaNbO_3_/ZnO	Convert mechanical energy into electrical energy	Excellent catalytic performance (degrade 97 % rhodamine B in 60 min), excellent piezoelectric ability (3.75 V electric output under ultrasonic vibrations)	[116]
		BaTiO_3_	Convert mechanical energy into electrical energy	Effective piezoelectric ability (113 mV electric output under 2.5 Hz vibrations), good mechanical properties (elastic modulus: 14.85 ± 4.85 kPa)	[114]
	Ultrasound	DT-Ag-CS^+^	Composite with ultrasound sensitizer TiO_2_	Good mechanical properties (specific surface area:76 m^2^/g), excellent biocompatibility (HUVECs and NIH-3T3 cell viability >90 %)	[128]
		HMME	Absorb ultrasonic energy and generate ROS	Sonodynamic antimicrobial activity (*P. gingivalis* CFU decreased 4.7 lg)	[263]
		TPP-TeV	Absorb ultrasonic energy and generate ROS	Excellent biocompatibility (L929 cells viability >85 %), superior antibacterial properties (>90 % inhibition against *P.gingivalis*)	[130]

Abbreviation: HPDLFs, human periodontal ligament fibroblasts; *P. gingivalis, Porphyromonas gingivalis*; *P. intermedia, Prevotella intermedia*; *A. actinomycetemcomitans, Aggregatibacter actinomycetemcomitans*; FLB, fluorobiprofen; BMP-2, bone morphogenetic protein-2; BMSCs, bone marrow mesenchymal stem cells; DLC, drug loading capacity; ALA, alpha-lipoic acid; MINO, minocycline; HGFs, human gingival fibroblasts; OBs, osteoblasts; GOx, glucose oxidase; rhBMP-2, recombinant human bone morphogenetic protein-2; BHT, 2,6-di-tert-butyl-4-methylphenol; CuTA, copper tannic acid coordination; PDLSCs, periodontal ligament stem cells; HUVECs, human umbilical vein endothelial cells; BE, baicalein; CHX, chlorhexidine acetate; PLGA, poly(lactic-co-glycolic acid); PGO, polydopamine-reduced grapheane oxide; PEDOT, poly(3,4-ethylenedioxythiophene); PSF, polydopamine-mediated silk microfiber; *F. nucleatum, Fusobacterium nucleatum;* NIR, near-infrared; *E. coli, Escherichia coli; C. albican, Candida albicans; S. aureus, Staphylococcus aureus;* hPDLSCs, human periodontal ligament stem cells; TBO, toluidine blue O; SDF-1, stromal cell derived factor-1; EE, encapsulation efficiency; LE, loading efficiency; PBS, phosphate-buffered saline; PDLCs, periodontal ligament cells; hPDLCs, human periodontal ligament cells; TMC, N,N,N-trimethyl chitosan; CS, chitosan; CMCS, carboxymethyl chitosan; PAMAM, poly(amidoamine); ZIF-8, zeolitic imidazolate frameworks-8; PEO, polyethylene oxide; TM, triglycerol monostearate; PEG, poly(ethylene glycol); DSPE-PEG, 1,2-distearoyl-sn-glycero-3-phosphoethanolamine-polyethylene glycol; PBA-PEI, phenylboronic acid-functionalized polyvinylimide; PCL-PLG, poly-ε-caprolactone- guanidinated-poly-ε-lysine; MSN, mesoporous silica; PLGA-TK-PEG, poly (lactic-co-glycolic acid)-thioketal-poly(ethylene glycol); Lip, liposome; MPN, metal–phenolic network; Alb, albumin; PPEMA-GelMA, polyphosphoester-methacrylic gelatin; CaAlg, calcium alginate; CAT, catalase; c-PVA, crosslinked polyvinyl alcohol; HTCC, N-(2-hydroxyl)propyl-3-trimethyl ammonium chitosan chloride; CO, carbon monoxide; MSNs-PMS, mesoporous silica nanocarriers core-shell porous microsphere; PPEM, polyphosphoester and minocycline hydrochloride; HQRB, hyperbranched quaternary ammonium-rose bengal; -SS-, disulfide bonds; Dex, dextran; BNP, bovine serum albumin nanoparticle; PHA, polydopamine-modified hydroxyapatite nanoparticles; AG, alginate/gelatin; GMPA, polypyrrole-decorated gelatin methacryloyl and oxidized hyaluronic acid; PEGPD, polyethylene glycol diacrylate-dithiothreitol-functional peptide module hydrogel; HMME, hematoporphyrin monomethyl ether; TPP-TeV, telluroviologen-anchored tetraphenylporphyrin derivative; DT-Ag-CS^+^, deposition of Ag modifying with the quaternary ammonium chitosan; PDLLA, poly(dl-lactide); PEO-PPO-PEO, poly(ethylene oxide-b-propylene oxide-b-ethylene oxide); PC, procyanidin; MPB, mesoporous prussian blue; BA, baicalein; PEG-b-PArg, poly(ethylene glycol)-b-polylarginine; C6, coumarin 6; Ce6, chlorin e6; PDA, polydopamine; NBP, nanobipyramid; PAA, polyacrylic acid.

### 6.1 Tailoring personalized medicine for microenvironment heterogeneity

The physiological microenvironment of periodontal disease exhibits significant heterogeneity among different patients. Future research can combine precise diagnosis with machine learning algorithms to design more intelligent and personalized drug delivery systems to adapt to the dynamic changes of the periodontal microenvironment. These variations include changes in ion concentration, enzyme activity, and local pH and redox state, all of which collectively impact the consistency and predictability of material responses. For instance, fluctuations in pH and variations in enzyme activity can lead to off-target drug release. [26,42] Additionally, alterations in the local redox state can influence the rate of drug release and bioavailability, thereby affecting treatment efficacy. [56,60] Here, high-precision biosensors offer new possibilities for integrating precise diagnosis and developing personalized materials with adjustable thresholds. For example, a new fluorescent probe can be used for detection and to quantify specific ROS. [264] The detection limit of this probe for H_2_O_2_ is as low as 9.43 × 10^−7^ M. Furthermore, the probe has a high selectivity for H_2_O_2_, and other metabolites have minimal interference with it, supporting specific diagnosis in complex biological environments. Similarly, there are also advanced sensors that can achieve high resolution monitoring of local pH and glucose levels. [265] Through these sensing technologies, the level of periodontal inflammation can be dynamically quantified, an individualized threshold database can be established and corresponding design materials can be designed. On the other hand, Machine Learning (ML) algorithms have opened up an innovative path for the personalized design and optimization of stimulus-response materials. The multi-stimulus response behavior of materials is not a simple linear superposition of a single stimulus, but rather presents complex patterns such as synergistic enhancement, antagonistic cancellation, or sudden response. [266] This nonlinear relationship stems from the complexity of the dynamically coupled molecular structure and multi-scale interactions within the materials. [266] The traditional method relies on high-cost experimental trial-and-error and is difficult to cope with the individualized demands. The ML model can efficiently analyze the spatial characteristics of material microstructures and capture their temporal response patterns in dynamic environments. [266,267] It significantly enhances the reliability of personalized design of stimulus-responsive materials.

### 6.2 Material biocompatibility and long-term safety assessment

Despite the broad application prospects of smart responsive materials, realizing their clinical value requires substantial efforts in material biocompatibility assessment and manufacturing processes. Current assessments of material biocompatibility primarily involve in vitro cell cultures and short-term animal models to evaluate the impact on cellular behavior and inflammatory responses. However, a comprehensive evaluation must also consider the long-term safety and efficacy of the materials, including their potential to induce chronic inflammation, fibrous capsule formation, or pose an infection risk. [[268], [269], [270]] Regulatory authorities like the Food and Drug Administration (FDA) emphasize complete lifecycle safety evidence for approving new materials. For example, in the FDA approval case of deflazacort, risk-controllable accelerated approval was achieved by establishing post-marketing studies despite lacking long-term toxicity data on major metabolites (like M − V). [271] This model offers a crucial reference for developing smart materials: incorporating long-term in vivo assessment metrics early on (*e.g.* 12-month subchronic toxicity, immunogenicity, metabolic pathway tracking) and integrating real-world data (RWD) to enhance the evidence chain. In future material safety optimization, prioritizing natural materials with clear metabolic pathways (such as liposomes, albumin) is advisable. Their degradation products can be cleared via hepatic and renal pathways, reducing accumulation toxicity risks. Systematically tracking materials' dynamic behaviors and cumulative effects within organisms is essential. Multi-dimensional assessments should examine organ pathology evolution, metabolic homeostasis disruption (such as toxic metabolite accumulation), and continuous immune system activation (such as anti-drug antibody production).

### 6.3 Manufacturing process optimization and clinical translation challenges

Large-scale production of smart responsive materials poses significant challenges. Transitioning from laboratory to industrial production requires maintaining the consistency and stability of materials, which can be influenced by numerous factors during synthesis, including temperature, pressure, and catalyst selection. Strict quality control measures must be implemented throughout the production process to ensure that each batch meets the desired standards. Emerging technologies such as microfluidic synthesis provide feasible solutions for this. They achieve precise control of nanoscale fluids through microchannel design and stabilize reaction conditions using a closed-loop feedback system. [272] In addition, microfluidic synthesis technology support continuous synthesis, ensuring the reproducibility of different batches. [273] Moreover, economic feasibility is another key consideration for large-scale production—how to reduce costs and improve efficiency while ensuring product quality. In this regard, intelligent manufacturing technologies may become the focus of future research, such as online monitoring systems based on machine vision. Machine vision is used for material quality control, featuring low hardware cost, low false alarm rate, high precision, and long-term stability. [274] This system can reduce manual intervention, lower costs and ensure process quality. In particular, the materials should comply with the quality control framework standards before being put into clinical application. According to 21 CFR Part 820, the FDA requires submission of material biocompatibility data (ISO 10993), 3 - month accelerated aging test results, and the risk assessment report (ISO 14971). [[275], [276], [277]] The standards of the European Medicines Agency (EMA) require the submission of pilot production data from multiple batches (traditionally three batches) to validate process robustness, alongside the completion of a Clinical Performance Study (CPR) to demonstrate the reproducibility of the device's functional performance. [278]

## CRediT authorship contribution statement

**Guang-Liang Su:** Writing – original draft, Visualization. **Yu-Jun Peng:** Writing – original draft, Visualization. **Hong-Ze Ruan:** Writing – original draft, Visualization. **Juan Cheng:** Writing – original draft, Visualization. **Tian Deng:** Supervision, Funding acquisition. **Yu-Feng Zhang:** Supervision, Funding acquisition.

## Declaration of competing interest

The authors declare that they have no competing interests.

## Data Availability

No data was used for the research described in the article.
